# The effect of vitamin D_3_ supplementation on the incidence of type 2 diabetes in healthy older adults not at high risk for diabetes (FIND): a randomised controlled trial

**DOI:** 10.1007/s00125-024-06336-9

**Published:** 2024-12-02

**Authors:** Jyrki K. Virtanen, Sari Hantunen, Niko Kallio, Christel Lamberg-Allardt, JoAnn E. Manson, Tarja Nurmi, Jussi Pihlajamäki, Matti Uusitupa, Ari Voutilainen, Tomi-Pekka Tuomainen

**Affiliations:** 1 University of Eastern Finland, Institute of Public Health and Clinical Nutrition, Kuopio, Finland; 2 University of Eastern Finland, School of Pharmacy, Kuopio, Finland; 3 University of Helsinki, Department of Food and Nutrition, Helsinki, Finland; 4 Department of Medicine, Brigham and Women’s Hospital Harvard Medical School, Boston, MA USA; 5 Department of Epidemiology, Harvard T.H. Chan School of Public Health, Boston, MA USA

**Keywords:** BMI, Experimental study, Older adults, Type 2 diabetes, Vitamin D_3_

## Abstract

**Aims/hypothesis:**

Vitamin D insufficiency is associated with an elevated risk of type 2 diabetes, but evidence from randomised trials on the benefits of vitamin D supplementation is limited, especially for average-risk populations. The Finnish Vitamin D Trial (FIND) investigated the effects of vitamin D_3_ supplementation at two different doses on the incidence of type 2 diabetes in a generally healthy older adult population.

**Methods:**

FIND was a 5 year randomised placebo-controlled, parallel-arm trial among 2271 male and female participants aged ≥60 years and ≥65 years, respectively, from a general Finnish population who were free of CVD or cancer and did not use diabetes medications. The study had three arms: placebo, 1600 IU/day of vitamin D_3_ or 3200 IU/day of vitamin D_3_. A non-study group statistician carried out sex-stratified simple randomisation in a 1:1:1 ratio, based on computerised random number generation. The participants, investigators and study staff were masked to group assignment. National health registries were used to collect event data. A representative subcohort of 505 participants had more detailed in-person investigations at months 0, 6, 12 and 24.

**Results:**

During the mean follow-up of 4.2 years, there were 38 (5.0%), 31 (4.2%) and 36 (4.7%) type 2 diabetes events in the placebo (*n*=760), 1600 IU/day vitamin D_3_ (*n*=744; vs placebo: HR 0.81; 95% CI 0.50, 1.30) and 3200 IU/day vitamin D_3_ (*n*=767; vs placebo: HR 0.92, 95% CI 0.58, 1.45) arms, respectively (*p*-trend=0.73). When the two vitamin D_3_ arms were combined and compared with the placebo arm, the HR was 0.86 (95% CI 0.58, 1.29). In the analyses stratified by BMI (<25 kg/m^2^ [*n*=813, number of type 2 diabetes events=12], 25–30 kg/m^2^ [*n*=1032, number of events=38], ≥30 kg/m^2^ [*n*=422, number of events=54]), the HRs in the combined vitamin D_3_ arms vs the placebo were 0.43 (95% CI 0.14, 1.34), 0.97 (0.50, 1.91) and 1.00 (0.57, 1.75), respectively (*p*-interaction <0.001). In the subcohort, the mean (SD) baseline serum 25-hydroxyvitamin D_3_ (25(OH)D_3_) concentration was 74.5 (18.1) nmol/l. After 12 months, the concentrations were 72.6 (17.7), 99.3 (20.8) and 120.9 (22.1) nmol/l in the placebo, 1600 IU/day vitamin D_3_ and 3200 IU/day vitamin D_3_ arms, respectively. In the subcohort, no differences were observed in changes in plasma glucose or insulin concentrations, BMI or waist circumference during the 24 month follow-up (*p* values ≥0.19).

**Conclusion/interpretation:**

Among generally healthy older adults who are not at high risk for diabetes and who have serum 25(OH)D_3_ levels that are sufficient for bone health, vitamin D_3_ supplementation did not significantly reduce the risk of developing diabetes.

**Trial registration:**

ClinicalTrials.gov NCT01463813.

**Graphical Abstract:**

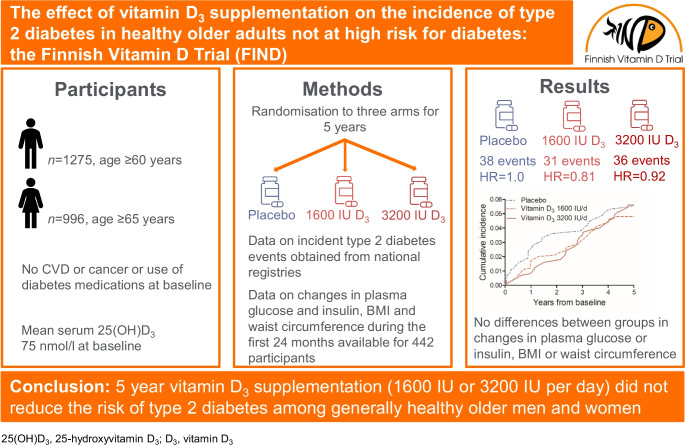

**Supplementary Information:**

The online version of this article (10.1007/s00125-024-06336-9) contains peer-reviewed but unedited supplementary material.


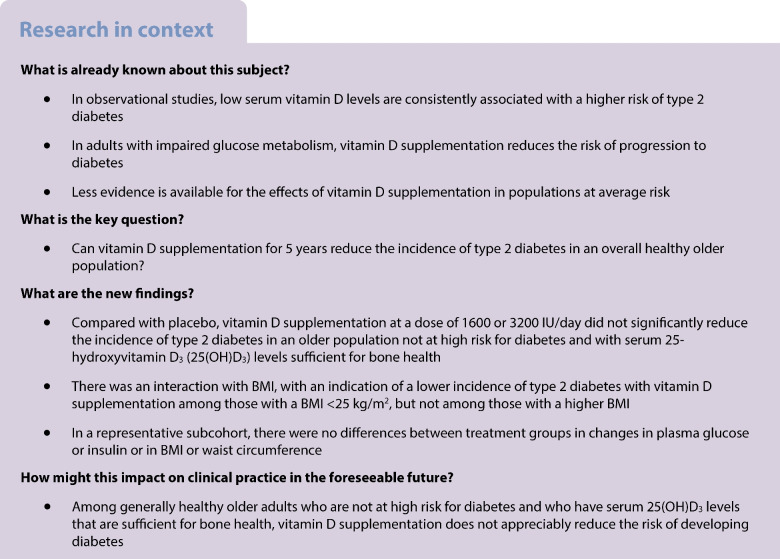


## Introduction

Between 1980 and 2021, the number of adults living with type 2 diabetes increased fivefold to 537 million, and the prevalence is predicted to keep rising globally [1]. Type 2 diabetes is a major cause of morbidity and mortality and poses a major societal burden related to healthcare services and lost productivity. Therefore, there is an urgent need to find approaches to reverse or at least slow down the rise in prevalence.

Vitamin D deficiency is common around the world [2] and it has been suggested that this may have unfavourable effects on glucose metabolism and risk of type 2 diabetes. Type 2 diabetes is characterised by dysfunction of pancreatic islet cells and consequent disturbances in insulin synthesis and secretion, and by insulin resistance in the peripheral tissues, resulting in impaired glucose metabolism or overt diabetes. Effects of vitamin D supplementation on glucose metabolism are plausible, because the physiologically active form of vitamin D, 1,25-dihydroxyvitamin D (1,25(OH)_2_D), is directly involved in the regulation of glucose metabolism by improving islet functioning and reducing insulin resistance [3]. Vitamin D deficiency has also been suggested to be associated with increased inflammation [3], which is part of the pathophysiology of type 2 diabetes [4]. In addition, vitamin D supplementation may favourably affect BMI and waist circumference [5]. Indeed, observational studies have consistently reported an association between vitamin D deficiency and increased risk of type 2 diabetes [6].

In short-term RCTs lasting an average of a few months, vitamin D supplementation has reduced fasting plasma glucose and insulin concentrations among participants without diabetes [7], but the benefits may be restricted to high-risk populations, that is, those with impaired glucose metabolism [8]. In addition, high-dose vitamin D supplementation has reduced the risk of progression of impaired glucose metabolism to diabetes [9, 10] and increased the likelihood of a reversal of hyperglycaemia to normoglycaemia [10], especially among participants without obesity [9] or those with a poorer vitamin D status at baseline [10]. In contrast, among average-risk participants, that is, those without impaired glucose metabolism, vitamin D supplementation has not affected the incidence of type 2 diabetes [7]. However, most studies that have reported the effects of supplementation on the incidence of type 2 diabetes among average-risk participants have lasted at most 12 months or have used low vitamin D doses [7]. Currently, there is little evidence from long-term RCTs whether moderate- or high-dose vitamin D supplementation could affect the incidence of type 2 diabetes among average-risk participants.

Here, we report the results from the Finnish Vitamin D Trial (FIND) regarding the effects of 5 year moderate- or high-dose vitamin D_3_ supplementation (1600 IU or 3200 IU daily vs placebo) on the incidence of type 2 diabetes, a prespecified tertiary outcome of the study, among generally healthy older men and women. In a subgroup of participants with available data, we also report the effects of supplementation on indices of glucose metabolism and on the major type 2 diabetes risk factors, BMI and waist circumference, during the first 2 years of the study.

## Methods

### Study population

FIND was conducted between September 2012 and October 2018. It was a double-blind, placebo-controlled RCT designed to investigate the effects of 5 year vitamin D_3_ supplementation on the incidence of major chronic diseases among generally healthy men and women from Finland [11]. The prespecified primary and secondary outcomes were CVD and cancer, and incidence of type 2 diabetes was a prespecified tertiary outcome.

The inclusion criteria were male participants aged ≥60 years and post-menopausal female participants aged ≥65 years without a history of CVD (including myocardial infarction, stroke, transient ischaemic attack, angina pectoris, coronary artery bypass grafting or percutaneous coronary intervention) or cancer (except non-melanoma skin cancer). The exclusion criteria were history of kidney stones, renal failure or dialysis, hypercalcaemia, hypo- or hyperparathyroidism, severe liver disease (cirrhosis) or sarcoidosis or other granulomatous diseases such as active chronic tuberculosis or Wegener’s granulomatosis; and use of vitamin D >800 IU/day or calcium >1200 mg/day from all supplemental sources combined (or, if taking, not willing to decrease or forego such use during the trial).

The original goal was to recruit 18,000 participants from three regions of eastern Finland (North Savo, North Karelia and Kainuu) [11], where, at the time of recruitment in 2012, 13% of the total population of about 500,000 people were men aged ≥60 years and 12% were women aged ≥65 years [12]. Biological sex was determined based on social security number and we aimed to recruit an approximately equal number of men and women. Data on ethnicity were not collected, because in eastern Finland the population is predominantly White in these age groups.

**Fig. 1:**
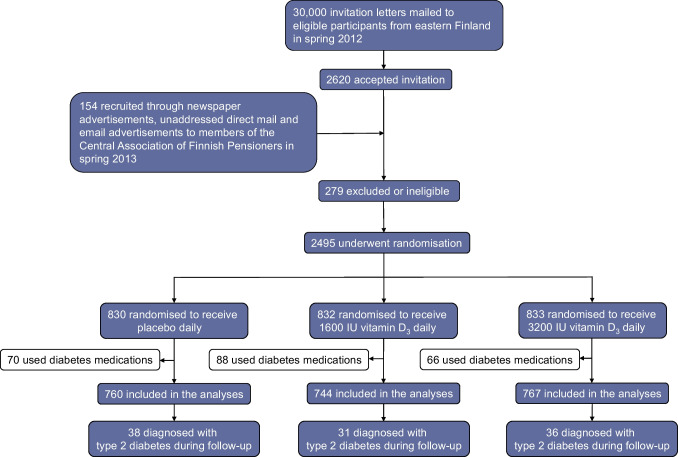
Participant flow chart

In total, the recruitment process yielded only 2495 participants, who were randomised to receive 1600 IU/day of vitamin D_3_, 3200 IU/day of vitamin D_3_ or placebo (Fig. 1) [11]. A random subcohort of 551 participants took part in the more detailed baseline examinations at the University of Eastern Finland, including blood sampling, with follow-up visits after 6, 12 and 24 months, respectively (see electronic supplementary material [ESM] Fig. 1) [11]. A non-study group statistician carried out sex-stratified simple randomisation in a 1:1:1 ratio, based on computerised random number generation. We did not have information on diabetes status at baseline, but we used self-reported use of diabetes medications as an exclusion criterion in the current analyses. Of the 2495 participants, 224 reported using diabetes medications at baseline; therefore, 2271 participants were included in the analyses of incident type 2 diabetes and 505 participants were included in the subcohort analyses (Fig. 1, ESM Fig. 1).

Pharmaceutical grade supplements (oral pills) were prepared specifically for the FIND trial by Galena Pharma (Kuopio, Finland). The pills contained either 0, 1600 IU or 3200 IU of vitamin D_3_. The pills were annually either mailed to the participants or given at study visits. Double-blinding was maintained throughout the study.

The participants completed questionnaires at baseline, after 12, 24 and 36 months and at the end of the trial at 60 months (an English translation of the questionnaire is available in the ESM) [11]. At baseline, 36 and 60 months the questionnaire also included a validated 142-item food frequency questionnaire. The final questionnaire included a question for assessing adherence to the study supplementation. Choices ranged from ‘<50%’ to ‘100% or almost 100%’.

BMI was calculated based on the weight and height reported in the baseline questionnaire. BMI based on self-reported data was used in the analyses that evaluated the effects of supplementation based on BMI. In the subcohort, waist circumference and weight and height were also measured by a study nurse during study visits. BMI based on these measurements was used in the analyses that investigated changes in BMI during follow-up in the subcohort.

In the subcohort of 504 participants with available blood samples, serum 25-hydroxyvitamin D_3_ (25(OH)D_3_) concentrations were measured using HPLC from samples collected at months 0, 6 and 12 [11, 13]. In a smaller subgroup of 60 participants, a direct competitive chemiluminescence immunoassay (Liaison 25 OH Vitamin D Total Assay, DiaSorin, Stillwater, MN, USA) was also used for serum 25(OH)D_3_ measurement from samples collected at months 0, 6, 12, 24 and 52 [11]. The serum 25(OH)D_3_ results were standardised against National Institute of Standards and Technology (NIST) reference values to generate representative values that were comparable to the HPLC results for statistical analyses [11].

All participants were free to withdraw from the study at any time without explanation. All participants provided written informed consent. The appropriate study approvals were obtained from the ethics committee of Kuopio University Hospital (#30/2010) and the Finnish Institute for Health and Welfare.

### Measurement of plasma glucose and insulin concentrations

Plasma glucose concentrations were assayed using the photometric hexokinase method (KoneLab 20XT, Thermo Fisher Scientific, Vantaa, Finland) and plasma insulin concentrations were measured using a chemiluminescence assay (DiaSorin Liaison, DiaSorin, Dietzenbach, Germany) from samples collected at baseline and at the 12 and 24 month study visits. HOMA-IR was calculated according to the previously published formula [14].

### Assessment of type 2 diabetes

The E11 codes from the ICD-10 (https://icd.who.int/browse10/2019/en) were used for diagnosis of type 2 diabetes mellitus. The codes were retrieved from two care notification registers, Hilmo for specialised healthcare [15] and Avohilmo for primary healthcare [16] (Finnish Institute for Health and Welfare, data agreement THL/523/5.05.00/2020). Causes of death were based on information provided by the Causes of Death Register (Statistics Finland, TK/1007/07.03.00/2022) [17]. The first date when the code E11 appeared in any of the registers was considered the date of diabetes diagnosis.

### Statistical analysis

Cox proportional hazards regression models adjusted for age and sex were used to predict the hazard of type 2 diabetes. Participants contributed follow-up time from randomisation until type 2 diabetes diagnosis, death, end of the 5 year follow-up or withdrawal from the study for personal reasons (*n*=412). The results are presented as HRs with 95% CIs. In the prespecified analyses, the two vitamin D arms were compared separately with the placebo arm. We also performed analyses that were not prespecified and should therefore be considered as exploratory. First, because of the low number of events, the effects of vitamin D supplementation were analysed after combining the two vitamin D arms. Second, potential latent effects of supplementation were investigated by excluding the type 2 diabetes events that occurred during the first 2 years of follow-up and by including the events that occurred during the post-supplementation follow-up period until 31 December 2021. In the subcohort with available data, changes in plasma glucose and insulin concentrations and HOMA-IR and in BMI and waist circumference between baseline and months 12 and 24 were analysed using linear mixed modelling. The logrank (Mantel–Cox) test was used to detect trends. Interactions were considered as multiplicative interactions. IBM SPSS version 29 (IBM, Armonk, NY, USA) was used for analyses. A two-sided *p* value of <0.05 was used to determine statistical significance. The *p* values were not adjusted for multiple comparisons.

## Results

The mean age of participants was 68.2 years and 57% were men. The baseline characteristics were similar between the study arms (Table 1). In the subcohort of 504 participants with available data, the mean±SD serum 25(OH)D_3_ concentration at baseline was 74.5±18.1 nmol/l (29.8±7.2 ng/ml, min.–max. 21.5–157.1 nmol/l; *p*-difference between arms=0.60), 9.3% (*n*=47) had a serum 25(OH)D concentration <50 nmol/l (20 ng/ml) and 49.0% (*n*=247) had a concentration >75 nmol/l (30 ng/ml). After 12 months, the mean±SD concentrations were 72.6±17.7 nmol/l (29.0±7.1 ng/ml, min.–max. 26.1–116.5 nmol/l), 99.3±20.8 nmol/l (39.7±8.3 ng/ml, min.–max. 59.0–159.1 nmol/l) and 120.9±22.1 nmol/l (48.4±8.8 ng/ml, min.–max. 70.8–194.9 nmol/l) in the placebo, 1600 IU/day vitamin D_3_ and 3200 IU/day vitamin D_3_ arms, respectively (*p*-difference <0.001). Data from a subgroup of 60 participants with serum 25(OH)D_3_ measurements at baseline and months 6, 12, 24 and 52 indicated that the differences between the arms were maintained throughout the 5 years [11].

**Table 1:** Baseline characteristics of participants

Characteristic	Overall (*n*=2271)	Placebo (*n*=760)	Vitamin D_3_ 1600 IU/day (*n*=744)	Vitamin D_3_ 3200 IU/day (*n*=767)
Female sex	996 (43.9)	350 (46.1)	317 (42.6)	329 (42.9)
Age (years)	68.2 (4.5)	68.1 (4.5)	68.1 (4.5)	68.3 (4.4)
Age group (years)				
60–64^a^	566 (24.9)	187 (24.6)	188 (25.3)	191 (24.9)
65–69	997 (43.9)	347 (45.7)	320 (43.0)	330 (43.0)
70–74	524 (23.1)	161 (21.2)	177 (23.8)	186 (24.3)
≥75	184 (8.1)	65 (8.6)	59 (7.9)	60 (7.8)
Employment status	(*n*=2247)	(*n*=754)	(*n*=736)	(*n*=757)
Full-time work	187 (8.3)	72 (9.5)	56 (7.6)	59 (7.8)
Part-time work	86 (3.8)	26 (3.4)	32 (4.3)	28 (3.7)
Unemployed	58 (2.6)	12 (1.6)	25 (3.4)	21 (2.8)
Retired	1901 (84.6)	641 (85.0)	615 (83.6)	645 (85.2)
Not working for other reasons	15 (0.7)	3 (0.4)	8 (1.1)	4 (0.5)
Leisure-time physical activity (h/week)^b^				
Light	13.2 (10.8) (*n*=1977)	13.3 (11.1) (*n*=652)	13.5 (11.2) (*n*=647)	12.8 (10.2) (*n*=678)
Heavy	5.7 (6.3) (*n*=1470)	5.6 (6.3) (*n*=487)	5.7 (6.1) (*n*=477)	5.9 (6.5) (*n*=506)
Smoking regularly^c^	782 (34.7) (*n*=2255)	257 (34.0) (*n*=755)	260 (35.3) (*n*=736)	265 (34.7) (*n*=764)
At least high school diploma	384 (17.0) (*n*=2260)	127 (16.8) (*n*=758)	135 (18.3) (*n*=739)	122 (16.0) (*n*=763)
Married	1693 (75.1) (*n*=2253)	571 (75.7) (*n*=754)	549 (74.4) (*n*=738)	573 (75.3) (*n*=761)
BMI (kg/m^2^)	26.8 (4.0) (*n*=2267)	26.9 (4.0) (*n*=758)	26.8 (4.0) (*n*=744)	26.7 (4.1) (*n*=765)
Alcohol intake (g/day)	7 (13) (*n*=2242)	8 (16) (*n*=754)	7 (11) (*n*=732)	7 (11) (*n*=756)
Vitamin D intake from diet (IU/day)	428 (316) (*n*=2242)	412 (264) (*n*=754)	456 (392) (*n*=732)	420 (280) (*n*=756)
Use of own vitamin D supplements				
Not at all	1499 (66.0)	483 (63.6)	501 (67.3)	515 (67.1)
200–400 IU/day	344 (15.1)	127 (16.7)	96 (12.9)	121 (15.8)
>400 to <800 IU/day	72 (3.2)	26 (3.4)	26 (3.5)	20 (2.6)
800 IU/day	356 (15.7)	124 (16.3)	121 (16.3)	111 (14.5)
Regular use of any medications	1472 (65.9) (*n*=2232)	512 (68.5) (*n*=747)	456 (62.6) (*n*=728)	504 (66.6) (*n*=757)
Self-rated health good or excellent	1379 (61.4) (*n*=2246)	440 (58.4) (*n*=753)	465 (63.5) (*n*=732)	474 (62.3) (*n*=761)

Data are *n* (%) or mean (SD)

^a^All were men in this age group

^b^Light activity was defined as gardening and other light outdoor activities; heavy activity was defined as physical exercise that causes sweating or heavy breathing

^c^Smoking regularly was defined as smoking almost every day during the last year

Among the 1471 participants who completed the last questionnaire, 95.4% (*n*=1404) reported using at least 80% of the pills during the study (*p*-difference between arms=0.73). Of those, 1091 (74.2% of all participants) reported taking all the study pills (*p*-difference=0.49).

### Incidence of type 2 diabetes

**Fig. 2:**
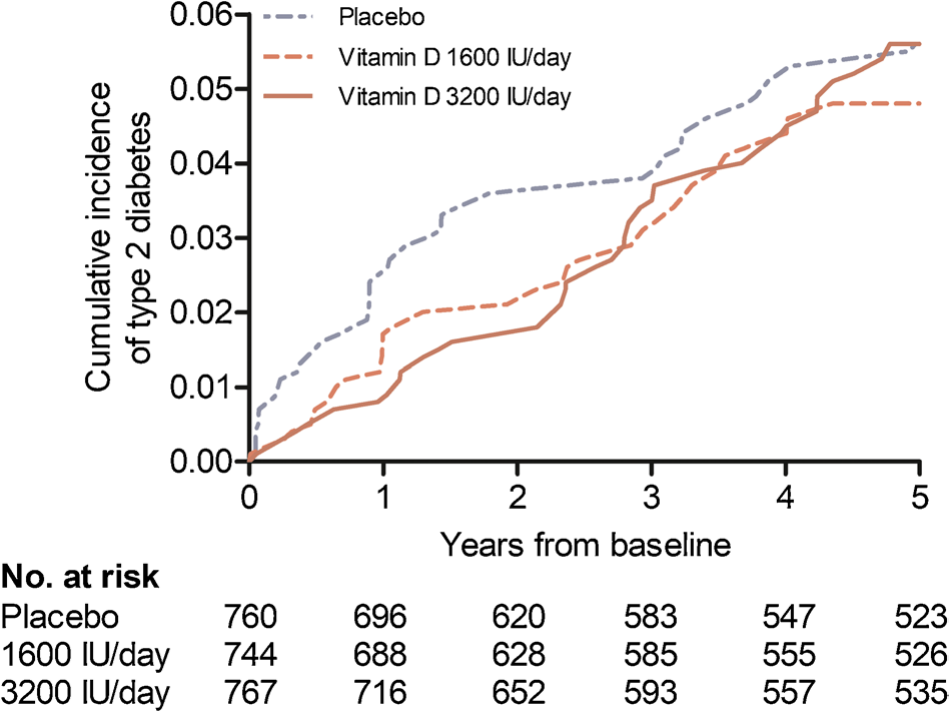
Cumulative incidence of type 2 diabetes in the three study arms during the 5 year supplementation period

During the mean follow-up of 4.2 years, 105 participants were diagnosed with type 2 diabetes. There were no statistically significant differences in event rates between the study arms (Table 2, Fig. 2). After excluding the 53 events that occurred during the first 2 years of follow-up, there was a trend towards an increased risk of type 2 diabetes with increasing vitamin D dose (*p*-trend=0.05; Table 2). There were no differences between the study arms in the analyses stratified by age and sex (Table 3). In the analyses stratified by the three BMI categories (<25, 25–30 and ≥30 kg/m^2^), the HRs and *p* values for interactions indicated a lower incidence of type 2 diabetes with vitamin D supplementation among those with a BMI <25 kg/m^2^, but the 95% CIs were wide and included 1 (Table 3).

**Table 2:** Incidence of type 2 diabetes during the 5 year supplementation period according to randomisation arm

Endpoint	Placebo (*n*=760)	Vitamin D_3_ 1600 IU/day (*n*=744)	*p* value	Vitamin D_3_ 3200 IU/day (*n*=767)	*p* value	*p* value for trend	Combined vitamin D arms vs placebo	*p* value
PY, *n*	3186.5	3179.7		3256.3				
Events, *n* (%)	38 (5.0)	31 (4.2)		36 (4.7)				
Rate per 100 PY (95% CI)	1.19 (0.87, 1.64)	0.97 (0.69, 1.38)		1.11 (0.80, 1.53)				
HR (95% CI)	1	0.81 (0.50, 1.30)	0.379	0.92 (0.58, 1.45)	0.708	0.731	0.86 (0.58, 1.29)	0.469
After exclusion of 53 participants with a type 2 diabetes diagnosis within the first 2 years of follow-up								
	(*n*=734)	(*n*=729)		(*n*=755)				
PY, *n*	3082.4	3115.3		3197.9				
Events, *n* (%)	12 (1.6)	16 (2.2)		24 (3.2)				
Rate per 100 PY (95% CI)	0.39 (0.22, 0.68)	0.51 (0.32, 0.84)		0.75 (0.50, 1.12)				
HR (95% CI)	1	1.31 (0.62, 2.77)	0.481	1.94 (0.97, 3.89)	0.060	0.050	1.62 (0.85, 3.11)	0.139

HRs (95% CIs) are adjusted for age and sex in the Cox proportional hazards regression model

PY, person-years

**Table 3:** Incidence of type 2 diabetes during the 5 year supplementation period according to subgroup and randomisation arm

Endpoint	No. of participants	Placebo	Vitamin D_3_ 1600 IU/day	Vitamin D_3_ 3200 IU/day	*p* value for trend	*p* value for interaction	Combined vitamin D arms vs placebo	*p* value for interaction
Men (events, *n*/*N* [%])	1275	26/410 (6.3)	16/427 (3.7)	23/438 (5.3)		0.862		0.791
HR (95% CI)		1	0.58 (0.31, 1.09)	0.83 (0.47, 1.46)	0.454		0.71 (0.43, 1.16)	
Women (events, *n*/*N* [%])	996	12/350 (3.4)	15/317 (4.7)	13/329 (4.0)				
HR (95% CI)		1	1.43 (0.67, 3.05)	1.14 (0.52, 2.50)	0.730		1.28 (0.65, 2.51)	
Age <67.4 years (events, *n*/*N* [%])	1139	22/393 (5.6)	16/375 (4.3)	18/371 (4.9)		0.756		0.555
HR (95% CI)		1	0.73 (0.38, 1.39)	0.83 (0.45, 1.55)	0.581		0.78 (0.46, 1.33)	
Age ≥67.4 years (events, *n*/*N* [%])	1132	16/367 (4.4)	15/369 (4.1)	18/396 (4.5)				
HR (95% CI)		1	0.94 (0.47, 1.91)	1.05 (0.54, 2.07)	0.922		1.00 (0.55, 1.82)	
BMI < median 26.2 kg/m^2^ (events, *n*/*N* [%])	1192	7/389 (1.8)	7/388 (1.8)	6/415 (1.4)		0.142		0.063
HR (95% CI)		1	0.97 (0.34, 2.76)	0.78 (0.26, 2.31)	0.679		0.87 (0.35, 2.18)	
BMI ≥ median 26.2 kg/m^2^ (events, *n*/*N* [%])	1075	31/369 (8.4)	24/356 (6.7)	29/350 (8.3)				
HR (95% CI)		1	0.77 (0.45, 1.31)	0.95 (0.57, 1.58)	0.855		0.86 (0.55, 1.34)	
BMI <25 kg/m^2^ (events, *n*/*N* [%])	813	6/258 (2.3)	2/263 (0.8)	4/292 (1.4)		0.002		<0.001
HR (95% CI)		1	0.31 (0.06, 1.51)	0.55 (0.15, 1.93)	0.345		0.43 (0.14, 1.34)	
BMI 25–30 kg/m^2^ (events, *n*/*N* [%])	1032	13/354 (3.7)	14/342 (4.1)	11/336 (3.3)				
HR (95% CI)		1	1.07 (0.50, 2.29)	0.87 (0.39, 1.95)	0.771		0.97 (0.50, 1.91)	
BMI ≥30 kg/m^2^ (events, *n*/*N* [%])	422	19/146 (13.0)	15/139 (10.8)	20/137 (14.6)				
HR (95% CI)		1	0.84 (0.43, 1.65)	1.16 (0.62, 2.18)	0.662		1.00 (0.57, 1.75)	

Values are HR (95% CI) adjusted for age and sex in the Cox proportional hazards regression model

Data for BMI were missing for four participants, of whom one was diagnosed with type 2 diabetes during the follow-up. Therefore, in the analyses stratified by BMI, the number of participants is 2267 and the number of events is 104

During the mean post-supplementation period of 3.3 years, 137 additional participants were diagnosed with type 2 diabetes, with 42, 48 and 47 in the placebo, 1600 IU/day vitamin D_3_ and 3200 IU/day vitamin D_3_ arms, respectively. There were no differences in event rates during the mean total follow-up period of 7.5 years or after the 53 events from the first 2 years of follow-up were excluded from the analyses (ESM Table 1, ESM Fig. 2).

### Plasma glucose and insulin concentrations, HOMA-IR, BMI and waist circumference in the subcohort

Table 4 presents mean values for fasting plasma glucose and insulin, HOMA-IR, BMI and waist circumference at baseline and 12 and 24 months in the subgroup with available data, as well as changes in these variables from baseline to 12 months and from 12 to 24 months. At baseline, mean BMI in those in the 3200 IU/day vitamin D_3_ arm was lower than in the other groups (*p*=0.047). However, there were no differences in the changes in any of the measurements between the three study arms (*p* values ≥0.19; Table 4) or when the two vitamin D supplementation groups were combined and compared with the placebo arm (*p* values ≥0.16; data not shown).

**Table 4:** Adjusted fasting plasma glucose and insulin concentrations, HOMA-IR, BMI and waist circumference at baseline, 12 and 24 months, and changes after baseline and 12 months according to randomisation arm

Measurement	Placebo	Vitamin D_3_ 1600 IU/day	Mean difference vitamin D_3_ 1600 IU vs placebo	Vitamin D_3_ 3200 IU/day	Mean difference vitamin D_3_ 3200 IU vs placebo	*p* value for arm	*p* value for time	*p* value for interaction
Plasma glucose, mmol/l						0.319	0.002	0.870
Baseline	5.68 (5.59, 5.77)	5.73 (5.64, 5.83)	0.05 (0.02, 0.08)	5.68 (5.59, 5.77)	−0.003 (−0.03, 0.02)			
Month 12	5.67 (5.56, 5.78)	5.75 (5.64, 5.86)	0.08 (0.05, 0.10)	5.64 (5.53, 5.86)	−0.03 (−0.06, −0.01)			
Month 24	5.76 (5.66, 5.85)	5.83 (5.73, 5.93)	0.07 (0.04, 0.10)	5.71 (5.62, 5.81)	−0.05 (−0.07, −0.02)			
Change in glucose, mmol/l						0.407	0.030	0.909
Month 12 vs baseline	−0.01 (−0.08, 0.07)	0.02 (−0.06, 0.10)	0.02 (0.02, 0.03)	−0.04 (−0.11, 0.04)	−0.05 (−0.06, −0.05)			
Month 24 vs month 12	0.08 (0.01, 0.16)	0.08 (0.004, 0.17)	0.001 (−0.0005, 0.003)	0.07 (−0.004, 0.15)	−0.01 (−0.011, −0.008)			
Plasma insulin, pmol/l						0.693	<0.001	0.700
Baseline	56.95 (51.25, 62.64)	59.38 (53.62, 65.21)	2.43 (2.01, 2.85)	57.50 (51.81, 63.27)	0.56 (0.14, 0.97)			
Month 12	60.49 (54.24, 66.74)	60.98 (54.52, 67.37)	0.42 (0.02, 0.83)	57.78 (51.43, 64.03)	−2.78 (−3.19, −2.36)			
Month 24	64.52 (57.85, 71.19)	67.78 (60.91, 74.59)	3.19 (2.78, 3.61)	62.37 (55.70, 69.03)	−2.22 (−2.57, −1.81)			
Change in insulin, pmol/l						0.375	0.116	0.593
Month 12 vs baseline	3.68 (−1.88, 7.57)	1.74 (−2.29, 5.76)	−1.94 (−2.01, −1.88)	0.28 (−3.61, 4.17)	−3.33 (−3.47, −3.26)			
Month 24 vs month 12	4.03 (−0.28, 8.40)	7.15 (2.64, 11.60)	3.13 (2.99, 3.19)	4.51 (0.21, 8.75)	0.49 (0.42, 0.56)			
HOMA-IR						0.678	<0.001	0.573
Baseline	2.13 (1.89, 2.36)	2.22 (1.98, 2.47)	0.09 (0.07, 0.12)	2.16 (1.92, 2.40)	0.03 (0.01, 0.06)			
Month 12	2.25 (2.00, 2.50)	2.27 (2.01, 2.52)	0.02 (−0.01, 0.04)	2.16 (1.91, 2.41)	−0.09 (−0.12, −0.06)			
Month 24	2.46 (2.17, 2.74)	2.62 (2.33, 2.91)	0.16 (0.13, 0.19)	2.35 (2.06, 2.63)	−0.11 (−0.14, −0.09)			
Change in HOMA-IR						0.187	0.032	0.604
Month 12 vs baseline	0.13 (−0.04, 0.29)	0.05 (−0.12, 0.22)	−0.07 (−0.08, −0.07)	−0.01 (−0.17, 0.16)	−0.13 (−0.134, −0.125)			
Month 24 vs month 12	0.21 (0.03, 0.39)	0.36 (0.16, 0.55)	0.15 (0.14, 0.15)	0.18 (−0.01, 0.36)	−0.03 (−0.04, −0.03)			
BMI, kg/m^2^						0.047	<0.001	0.583
Baseline	27.61 (26.96, 28.26)	27.78 (27.12, 28.45)	0.17 (0.15, 0.20)	26.71 (26.05, 27.37)	−0.90 (−0.92, −0.88)			
Month 12	27.52 (26.88, 28.17)	27.56 (26.91, 28.22)	0.04 (0.02, 0.07)	26.51 (25.86, 27.16)	−1.01 (−1.03, −0.99)			
Month 24	27.47 (26.83, 28.12)	27.51 (26.85, 28.17)	0.04 (0.01, 0.06)	26.51 (25.86, 27.17)	−0.96 (−0.98, −0.93)			
Change in BMI, kg/m^2^						0.505	0.023	0.506
Month 12 vs baseline	−0.08 (−0.22, 0.05)	−0.21 (−0.35, −0.07)	−0.13 (−0.14, −0.12)	−0.20 (−0.33, −0.06)	−0.12 (−0.12, −0.11)			
Month 24 vs month 12	−0.04 (−0.16, 0.08)	−0.05 (−0.17, 0.08)	−0.01 (−0.02, −0.001)	0.002 (−0.12, 0.12)	0.04 (0.03, 0.05)			
Waist circumference, cm						0.064	<0.001	0.307
Baseline	95.71 (93.98, 97.44)	97.23 (95.46, 99.01)	1.43 (0.50, 2.37)	94.11 (92.36, 95.86)	−1.68 (−2.60, −0.76)			
Month 12	95.94 (94.21, 97.67)	96.63 (94.86, 98.41)	0.60 (−0.33, 1.53)	94.03 (92.29, 95.78)	−1.99 (−2.91, −1.07)			
Month 24	94.91 (93.24, 96.59)	95.90 (94.18, 97.62)	0.90 (−0.03, 1.83)	93.09 (91.40, 94.78)	−1.91 (−2.83, −0.98)			
Change in waist circumference, cm						0.606	0.005	0.192
Month 12 vs baseline	0.23 (−0.42, 0.88)	−0.59 (−1.26, 0.08)	−0.82 (−0.85, −0.79)	−0.09 (−0.74, 0.57)	−0.32 (−0.35, −0.30)			
Month 24 vs month 12	−1.05 (−1.47, −0.63)	−0.69 (−1.12, −0.26)	0.35 (0.32, 0.38)	−0.95 (−1.36, −0.53)	0.09 (0.07, 0.12)			

Values are mean (95% CI) adjusted for age and sex in the linear mixed model

The numbers of participants at baseline and 12 and 24 months were 501, 482 and 451, respectively, for plasma glucose measurements; 501, 482 and 442, respectively, for plasma insulin and HOMA-IR analyses; 505, 487 and 455, respectively, for BMI; and 505, 487 and 454, respectively, for waist circumference

## Discussion

In this 5 year RCT among generally healthy and vitamin D-sufficient Finnish older adults, moderate- and high-dose vitamin D_3_ supplementation did not reduce the incidence of type 2 diabetes and, based on the subcohort data, did not have an effect on plasma glucose or insulin concentrations, HOMA-IR, BMI or waist circumference compared with placebo. The effects were similar in both men and women, indicating that sex does not affect the impact of vitamin D supplementation on glucose metabolism.

Observational studies have consistently reported that low concentrations of serum 25(OH)D, an established biomarker for vitamin D status, are associated with a higher risk of type 2 diabetes [6]. However, evidence from observational studies alone cannot be used to reliably establish causality because of potential confounding factors. For example, low serum 25(OH)D concentrations are often associated with higher BMI and adiposity and with lower physical activity levels, which are major risk factors for type 2 diabetes.

Evidence from experimental studies suggests that vitamin D supplementation may have different effects on type 2 diabetes incidence based on individuals’ glycaemic status. In individuals with impaired glucose metabolism, moderate- or high-dose vitamin D supplementation (≥1000 IU/day) has been shown to reduce the incidence of type 2 diabetes [9]. A recent systematic review carried out during development of the US Endocrine Society’s clinical practice guidelines for vitamin D supplementation concluded that there is moderate-certainty evidence that vitamin D supplementation decreases the progression of impaired glucose metabolism to diabetes in adults [18]. The guidelines suggest empirical vitamin D supplementation (i.e. higher doses than recommended by dietary reference intakes and implemented without testing for serum 25(OH)D concentrations) in addition to lifestyle modification for the prevention of type 2 diabetes among people at high risk [18]. In addition, a recent individual participant data meta-analysis of three RCTs that were specifically designed for diabetes prevention and included participants with impaired glucose metabolism found that vitamin D supplementation reduced the incidence of type 2 diabetes by 15% and increased the likelihood of regression to normoglycaemia by 30% compared with placebo [10]. The decrease in type 2 diabetes incidence was greatest among those who achieved and maintained the highest serum 25(OH)D concentrations of at least 125 nmol/l during the studies. This is close to the mean 120 nmol/l concentration that was achieved in the 3200 IU/day group in our trial. However, our findings in an average-risk population rather suggest a potentially increased risk of type 2 diabetes with increasing vitamin D dose in the analyses where the type 2 diabetes events from the first 2 years were excluded. These participants may already have had undiagnosed type 2 diabetes or at least impaired glucose metabolism at baseline (see the limitations paragraph below), so excluding them from the analyses may have revealed the true effect of vitamin D supplementation on type 2 diabetes incidence. Because the increase in risk did not persist in the post-supplementation period, the effect may be temporary and reversible when high-dose vitamin D supplementation is stopped. However, as the number of events was modest, the *p* values were not corrected for multiple comparisons and the subcohort analyses did not show unfavourable effects of vitamin D on plasma glucose or insulin concentrations or on body size, the possibility of a chance finding cannot be excluded and therefore caution is warranted when interpreting the finding of increased type 2 diabetes risk. Similarly, although our finding of a possibly lower incidence of type 2 diabetes among leaner participants agrees with previous observations [8], this result should also be considered exploratory because of the low number of events and consequent wide 95% CIs. However, we have previously reported that, compared with those with higher BMIs, those with a BMI <25 kg/m^2^ had the greatest mean increase in serum 25(OH)D_3_ concentrations after 12 months of vitamin D supplementation [11]. This may be attributed to the suppressed conversion of vitamin D to 25(OH)D in individuals with obesity [19]. Also, because vitamin D supports pancreatic beta cell function by binding to beta cell receptors and regulating calcium flux through beta cells, which is important for insulin secretion [20], vitamin D may be of greater benefit in leaner people, who may have insulin deficiency rather than insulin resistance, than in those with obesity. These factors could potentially contribute to the differences in effects observed in those with lower vs higher BMI.

There is little prior experimental research data from long-term RCTs with populations at an average risk for type 2 diabetes, and such studies used much lower vitamin D doses than our study. The Women’s Health Initiative included 33,951 post-menopausal women with a mean age of 62 years [21]. In a subgroup of participants, 61% had serum 25(OH)D concentrations <50 nmol/l. The participants were randomised to receive either 400 IU/day of vitamin D_3_ plus 1000 mg/day of calcium or placebo for a median of 7 years. No difference in the incidence of type 2 diabetes was observed between the groups (HR 1.01, 95% CI 0.94, 1.10). A similar lack of effect was reported in the RECORD trial, which included 4829 men and women with a mean age of 77 years who received 800 IU/day of vitamin D_3_, 1000 mg/day of calcium, both or placebo for 2–5 years (HR 1.11, 95% CI 0.77, 1.62 for the vitamin D group vs placebo group) [22]. In a small subgroup of 60 study participants, the mean baseline 25(OH)D concentration was 38 nmol/l. In both studies, diabetes status at baseline and the ascertainment of type 2 diabetes diagnosis during follow-up were based on self-report, which may affect the accuracy of diagnosis.

The strengths of this study include the population-based recruitment, long-follow-up and collection of data on incident type 2 diabetes events from national health registries. The use of national health registers may also be a limitation, because we may have missed some type 2 diabetes diagnoses that would have been detected by, for example, blood glucose monitoring one to two times per year. This may have reduced the power to detect an effect. Another major strength of this study was the use of both moderate and high vitamin D_3_ doses, which enabled investigation of any dose–response relationships. However, this was hampered by the modest number of type 2 diabetes events during the study period and by the fact that serum samples and other more detailed examinations were available only for the subcohort. For example, we could not investigate whether vitamin D-deficient participants would have benefited from supplementation, as data on serum 25(OH)D_3_ concentrations were available only for the subcohort. However, as <10% of subcohort participants had vitamin D insufficiency (serum 25(OH)D_3_ concentrations <50 nmol/l), it is unlikely that we would have had enough power for such analyses in the whole study population either. The differences in achieved serum 25(OH)D_3_ concentrations between the three groups may also have been too small to see major differences in the outcomes. Other limitations are that the study was not designed or powered for diabetes prevention and we did not have information on participants’ diabetes history at baseline. Although the self-reported use of type 2 diabetes medications as a proxy for disease status should reliably exclude those who have diabetes, we may have missed participants who did have diabetes but who were not treated with medication. As the participants in our study were White older men and women with sufficient baseline 25(OH)D_3_ concentrations, caution is needed when generalising the results to other age groups, racial and ethnic groups, and groups with different vitamin D levels. We also have no data on the representativeness of the FIND cohort demographics (e.g. education level, marital status, socioeconomic factors) compared with the whole source population.

In conclusion, our findings do not suggest benefits of long-term moderate- or high-dose vitamin D_3_ supplementation for incidence of type 2 diabetes or glucose metabolism or body size among generally healthy older vitamin D-sufficient men and women who were not at high risk for type 2 diabetes. In such populations without a high risk for developing type 2 diabetes, for any intervention to show a benefit in terms of preventing type 2 diabetes development, an impractically large study population would most likely be needed. However, our study results do not exclude the possibility that high-dose vitamin D supplementation could be beneficial among vitamin D-deficient populations with an average risk for type 2 diabetes. Currently, such research data are lacking.

## Supplementary Information

Below is the link to the electronic supplementary material.

ESM (PDF 1.01 MB)

## Data Availability

The data are not openly available because they contain sensitive personal information relating to the participants that cannot be completely anonymised. However, the analytical code used for the current study can be made available on reasonable request, and the data are open for potential research collaboration by contacting the corresponding author.

## Abbreviations

25(OH)D_3_: 25-hydroxyvitamin D_3_
FIND: Finnish Vitamin D Trial

## Authors’ relationships and activities

JKV reports receiving travel support to attend vitamin D research meetings from Abiogen Pharma. CL-A reports involvement in the Nordic Nutrition Recommendations 2023 as a member of the Systematic Review Centre, and in European Food Safety Authority procurement and preparatory work for the 2023 update of the tolerable upper intake levels for vitamin D. JEM reports receiving grants from the National Institutes of Health during the conduct of the study, and grants from the National Institutes of Health and from Mars Edge outside the submitted work. MU reports receiving a grant from Orion outside the submitted work. The authors declare that there are no other relationships or activities that might bias, or be perceived to bias, their work.
