# Supplementary material for: The effect of vitamin D3 supplementation on the incidence of type 2 diabetes in healthy older adults not at high risk for diabetes (FIND): a randomised controlled trial
Source: Diabetologia. 2024 Dec 2;68(4):715–26. doi: 10.1007/s00125-024-06336-9 (PMC11950068; doi:10.1007/s00125-024-06336-9)
Supplement: Supplementary file 1 — ESM (PDF 1.01 MB) [file 125_2024_6336_MOESM1_ESM.pdf]

The effect of vitamin D_3 supplementation on the incidence of type 2 diabetes in healthy older adults not at high risk for diabetes – the Finnish Vitamin D Trial (FIND): a randomized controlled trial

Jyrki K. Virtanen, Sari Hantunen, Niko Kallio, Christel Lamberg-Allardt, JoAnn E. Manson, Tarja Nurmi, Jussi Pihlajamäki, Matti Uusitupa, Ari Voutilainen, Tomi-Pekka Tuomainen

ELECTRONIC SUPPLEMENTARY MATERIAL

ESM Table 1 Incidence of type 2 diabetes during the 5-year supplementation period and extended post supplementation follow-up period until the end of year 2021 according to randomization arm

Endpoints	Placebo (n=760)	Vitamin D_3 1600 IU/day (n=744)	*p* value	Vitamin D_3 3200 IU/day (n=767)	*p* value	*p* value for trend	Combined vitamin D arms vs. placebo	*p* value
PY, *n*	5957.0	5933.5		6055.7				
Events, *n* (%)	80 (10.5)	79 (10.6)		83 (10.8)				
Rate per 100 PY (95% CI)	1.34 (1.08–1.67)	1.33 (1.07–1.66)		1.37 (1.11–1.70)				
Hazard ratio (95% CI)	1	0.97 (0.71–1.33)	0.854	1.01 (0.74–1.37)	0.948	0.900	0.99 (0.76–1.30)	0.946

After exclusion of 53 participants with type 2 diabetes diagnosis within the first 2 years of follow-up

	Placebo (n=734)	Vitamin D_3 1600 IU/day (n=729)	*p* value	Vitamin D_3 3200 IU/day (n=755)	*p* value	*p* value for trend	Combined vitamin D arms vs. placebo	*p* value
PY, *n*	5752.1	5815.6		5942.7				
Events, *n* (%)	54 (7.4)	64 (8.8)		71 (9.4)				
Rate per 100 PY (95% CI)	0.94 (0.72–1.22)	1.10 (0.86–1.40)		1.19 (0.95–1.51)				
Hazard ratio (95% CI)	1	1.16 (0.81–1.67)	0.423	1.28 (0.90–1.83)	0.169	0.155	1.22 (0.89–1.68)	0.215

HRs (95% CIs) are adjusted for age and sex in the Cox proportional hazards regression model.
PY, person-years

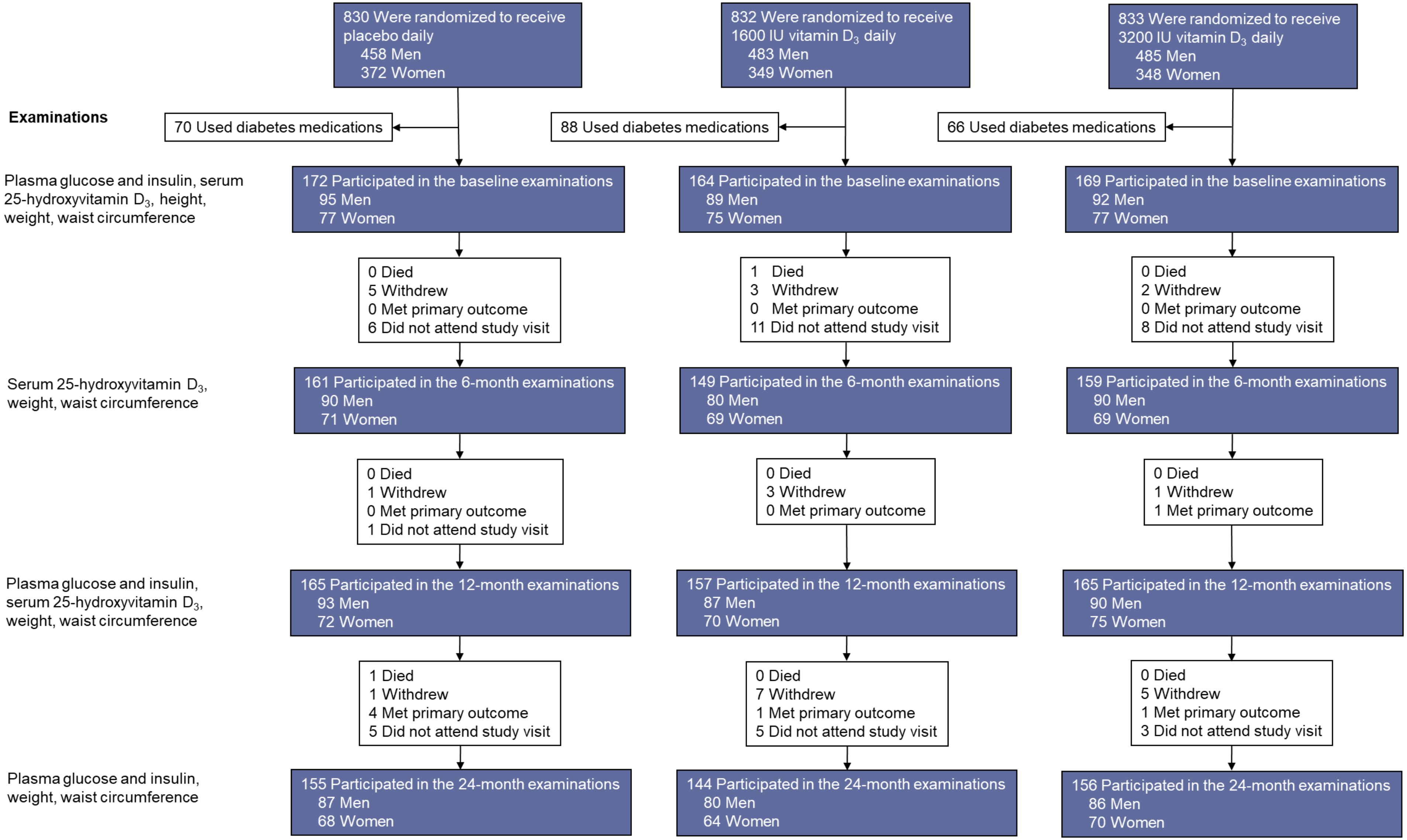

ESM Fig. 1 Number of participants in the subcohort study visits in the three supplementation arms and the examinations done on each visit

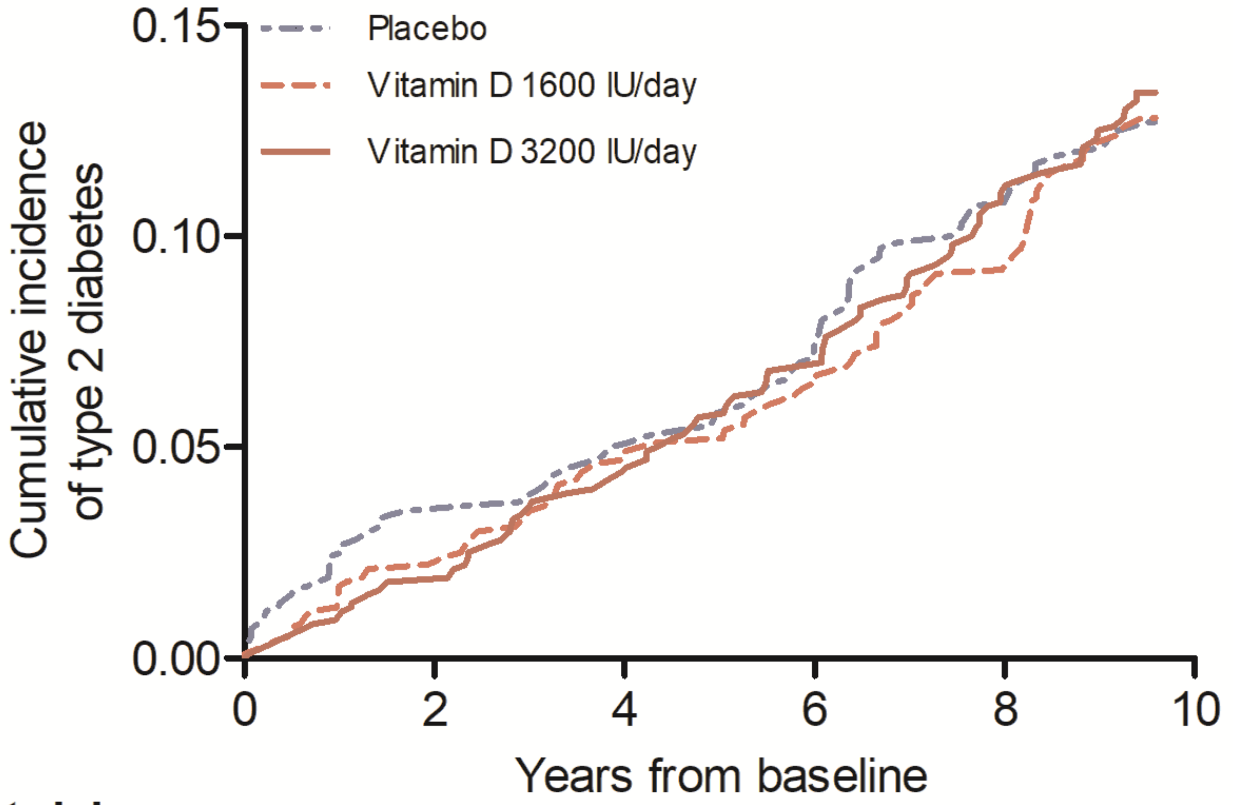

No. at risk

Placebo	760	645	583	554	519	453
1600 IU/day	744	647	593	561	533	448
3200 IU/day	767	670	591	563	527	453

ESM Fig. 2 Incidence of type 2 diabetes in the three study arms during the 5 year supplementation period and extended post supplementation follow-up period until the end of year 2021

UNIVERSITY OF
EASTERN FINLAND

Finnish Vitamin D Trial

This is an unofficial English translation of the original study forms that were in Finnish

PLEASE ANSWER THE FOLLOWING QUESTIONS:

Have you had

- a heart attack? No ☐ Yes ☐
- a stroke or a transient ischemic attack (TIA)? No ☐ Yes ☐

Have you undergone

- heart bypass surgery? No ☐ Yes ☐
- coronary angioplasty? No ☐ Yes ☐

Have you ever been diagnosed with

- cancer (other than basal cell carcinoma of the skin)? No ☐ Yes ☐
- kidney disease (kidney stones, insufficiency, etc.)? No ☐ Yes ☐
- liver disease? No ☐ Yes ☐
- elevated blood calcium levels (hypercalcemia)? No ☐ Yes ☐
- sarcoidosis? No ☐ Yes ☐
- overactive or underactive parathyroid glands? No ☐ Yes ☐
  (thyroid overactivity or underactivity does not disqualify participation)

Do you take

- vitamin D supplements over 20 µg/day? No ☐ Yes ☐
  (consider all vitamin D-containing supplements)
- calcium supplements over 1200 mg/day? No ☐ Yes ☐

If you answered "No" to all of the above, you may be eligible for the study. In that case, please follow the instructions below.

If you answered "Yes" to any question, unfortunately, you are not eligible for the study, and you do not need to return this form.

<u>INSTRUCTIONS:</u>

- Carefully read the separate "Information for Participants" document.
- Answer the questions above.
- If you answered "No" to all the questions and are willing to participate in the study, please fill out this booklet and return it to us in the enclosed prepaid return envelope.

Please also ensure that you have signed both consent forms included in this booklet.

Thank you for completing the forms. You will be informed of your inclusion in the study and receive any necessary study materials by the end of May 2012.

CONSENT FORM 1

Please complete both consent forms included in this booklet. One copy will be returned to you, signed, along with the study materials.

Study on the Effects of Vitamin D on the Prevention of Cardiovascular Diseases and Cancers (Finnish Vitamin D Trial – FIND)

I, ____, have been invited to participate in the above-mentioned study, which aims to investigate the effects of vitamin D supplementation in preventing common chronic diseases, such as cardiovascular diseases and cancers. I have read and understood the written information provided about the study. From the information sheet, I have received sufficient details regarding the study, the collection, processing, and disclosure of information. I have had the opportunity to contact the study nurses and researchers to discuss the study before signing the consent form.

I have had enough time to consider my participation in the study. I have been adequately informed about my rights, the study's purpose, and its execution, as well as the benefits and risks involved. I also give permission to obtain information about my health from national registers and to combine this data with the information gathered in the study.

If you are willing to take tablets that may contain vitamin D for five years and complete annual questionnaires, please answer the following three questions:

1) I consent to giving a blood sample upon request: YES ☐ NO ☐

2) I consent to being invited to a study visit in Kuopio: YES ☐ NO ☐

3) I would like to complete the annual questionnaires online in the following years: YES ☐ NO ☐

If you answered NO to question 2 but are still willing to take the study supplement and complete the annual questionnaires, either on paper or online, you may participate in the study.

I have not been coerced or persuaded into participating in this study. I understand that my participation is voluntary, and I am aware that I can withdraw my consent at any time without providing a reason. I also understand that withdrawing my consent will not affect my treatment or care in any way. I am aware that my personal information will be treated confidentially and will not be disclosed to third parties. I also understand that if I withdraw from the study, the data and samples collected up to the point of withdrawal will still be used as part of the study material.

Consent Form 1, Page 2

_______________ ________________________ |__|__|__|__|__|__|__|__|__|__|__|
First Name **Last Name** **Personal ID Number**

____
Street Address

|__|__|__|__|__| ____
Postal Code **City/Town**

____
Phone Number

____
Email Address

_______________ ____
Date **Signature**

Consent received by:

____________________________ ______________ _______________________________
Receiver of Consent **Date** **Signature**

One of the signed consent forms and a copy of the study information will be archived at the research center. The other signed consent form will be returned to the participant along with the study materials.

CONSENT FORM 2

Please complete both consent forms included in this booklet. One copy will be returned to you, signed, along with the study materials.

Study on the Effects of Vitamin D on the Prevention of Cardiovascular Diseases and Cancers (Finnish Vitamin D Trial – FIND)

I, ____, have been invited to participate in the above-mentioned study, which aims to investigate the effects of vitamin D supplementation in preventing common chronic diseases, such as cardiovascular diseases and cancers. I have read and understood the written information provided about the study. From the information sheet, I have received sufficient details regarding the study, the collection, processing, and disclosure of information. I have had the opportunity to contact the study nurses and researchers to discuss the study before signing the consent form.

I have had enough time to consider my participation in the study. I have been adequately informed about my rights, the study's purpose, and its execution, as well as the benefits and risks involved. I also give permission to obtain information about my health from national registers and to combine this data with the information gathered in the study.

If you are willing to take tablets that may contain vitamin D for five years and complete annual questionnaires, please answer the following three questions:

1) I consent to giving a blood sample upon request: YES ☐ NO ☐

2) I consent to being invited to a study visit in Kuopio: YES ☐ NO ☐

3) I would like to complete the annual questionnaires online in the following years: YES ☐ NO ☐

If you answered NO to question 2 but are still willing to take the study supplement and complete the annual questionnaires, either on paper or online, you may participate in the study.

I have not been coerced or persuaded into participating in this study. I understand that my participation is voluntary, and I am aware that I can withdraw my consent at any time without providing a reason. I also understand that withdrawing my consent will not affect my treatment or care in any way. I am aware that my personal information will be treated confidentially and will not be disclosed to third parties. I also understand that if I withdraw from the study, the data and samples collected up to the point of withdrawal will still be used as part of the study material.

Consent Form 1, Page 2

________________ _________________________ |___|___|___|___|___|___|___|___|___|___|___|

First Name **Last Name** **Personal ID Number**

____

Street Address

|___|___|___|___|___| ____

Postal Code **City/Town**

____

Phone Number

____

Email Address

________________ ____

Date **Signature**

Consent received by:

_____________________________ ______________ ________________________________

Receiver of Consent **Date** **Signature**

One of the signed consent forms and a copy of the study information will be archived at the research center. The other signed consent form will be returned to the participant along with the study materials.

Date of completion: |___|___| . |___|___| 201X

FIND Study, QUESTIONNAIRE

Finnish Vitamin D Trial

Please read each question carefully and choose the answer that best describes you by circling the appropriate option. Mail the completed form in the enclosed envelope, for which postage has been prepaid. **If you have any difficulties completing this form, you can contact the researchers at the phone numbers 02944 54xxx or 02944 54xxx, or study nurse x.x at 02944 54xxx on weekdays from 12 to 15, or by email at d-vitamiini@uef.fi.**

A. BACKGROUND INFORMATION (circle the most appropriate option or provide your answer on the line)

1. Weight: |___|___|___| kg **2. Height:** |___|___|___| cm

3. What is your marital status?

1 Single, never married
2 Married or cohabiting
3 Divorced
4 Widowed

4. Household size (including yourself)? |____|____| persons

5. What is your education level?

1 Some elementary school
2 Elementary school or part of middle school
3 Middle school or part of high school
4 Middle school and vocational training
5 High school diploma
6 High school diploma and another degree
7 Academic degree

6. What is your current employment status?

01 Full-time work (or on vacation, leave, etc.)
02 Part-time work
03 Laid off (reduced work week or fully laid off)
04 Unemployed, since |__|__|__|__|
05 On unemployment pension, since |__|__|__|__|
06 On partial retirement, since |__|__|__|__|
07 Early retirement, since |__|__|__|__|
08 On disability pension, since |__|__|__|__|
09 Retired (old-age pension), since |__|__|__|__|
10 Out of work for other reasons

7. What is/was your most recent occupation?

1 Office work (sedentary work)
2 Light indoor work (standing work or similar)
3 Physically heavy indoor work (factory work or similar)
4 Light outdoor work
5 Physically heavy outdoor work

B. SUN EXPOSURE

Consider the **past 12 months** when answering these questions. If you are completing the form in the spring, think about the summer of the previous year. If you are completing it in the fall, consider the current year's summer.

8. Have you traveled to sunny destinations abroad in the past 12 months?

0 No (skip to question 10)
1 Yes

9. If you answered yes, how long, where, and when?

Total number of sunny vacation days in the past 12 months: |___|___|___| days

Most recent destination:
1 Mediterranean
2 Canary Islands
3 Asia or the Caribbean
4 Other

Start date of the most recent sunny vacation: |___|___| . |___|___| 201___

10. Have you vacationed or gone skiing in Lapland or in foreign ski resorts in the past 12 months?

0 No (skip to question 12)
1 Yes

11. If you answered yes, how long, where, and when?

Total number of vacation days in the past 12 months: |___|___|___| days

Most recent destination:
1 Lapland
2 Central Europe
3 Other

Start date of the most recent ski vacation: |___|___| . |___|___| 201___

12. Have you used a tanning bed in the past year?

0 No (skip to question 14)
1 Yes

13. If you answered yes, how many times? |___|___| times

14. Estimate how many hours per day you spent outdoors during June-August 2011.

On weekdays: |____|____| hours and on weekends: |____|____| hours

15. When you are outdoors, how do you react to sunlight?

1 I try to avoid direct sunlight
2 I am occasionally in the sun
3 I sunbathe to get a tan

16. When you are outdoors in sunny and warm weather, how do you dress?

1 I wear long-sleeved shirts and long pants or skirts
2 I wear short-sleeved shirts and long pants/skirts, or long-sleeved shirts and shorts/short skirts
3 I wear short-sleeved shirts and shorts/short skirts
4 I wear light beachwear or a swimsuit

17. Do you usually use sunscreen with a sun protection factor (SPF) during the summer?

0 No (skip to question 19)
1 Yes

18. If you answered yes, what SPF does the sunscreen you usually use have? |___|___|

C. PHYSICAL ACTIVITY

19. My work includes light physical activity for |___|___| hours per week.

20. My work includes heavy physical activity, during which I sweat or become out of breath for |___|___| hours per week.

21. I engage in light physical activity or physical utility during my free time (yard work and other outdoor chores, etc.) for |___|___| hours per week.

22. I engage in heavy physical activity or physical utility during my free time, during which I sweat or become out of breath for |___|___| hours per week.

D. SMOKING

23. Have you ever smoked regularly (almost every day for at least a year)?

0 No (skip to question 26)
1 Yes, for a total of |____|____| years

24. Have you smoked in the last month (30 days)?

0 No (skip to question 26)
1 1 Yes

25. If you have smoked daily/almost daily in the last month (30 days), how much do you smoke or did you smoke on average per day (circle)?

	Not at all	1-5	6-19	20 or more
Cigarettes	0	1	2	3
Cigars	0	1	2	3
Pipe	0	1	2	3
E-cigarettes	0	1	2	3
Snuff	0	1	2	3

N.B. The food frequency questionnaire was included in the form at months 0, 36 and 60.

E. FOOD FREQUENCY QUESTIONNAIRE

UNIVERSITY OF EASTERN FINLAND

The purpose of this survey is to get an overview of your eating habits. When you fill out this form, think about **the past 12 months**. If you do not cook or participate in purchasing food, it is good to fill out the survey with the family member who does the cooking. Before you start filling out the form, familiarize yourself with it by looking through it.

In the following questions about eating, circle the one option that best describes your habits, except in question 26, where you can choose as many options as needed.

26. Which of the following meals do you eat on a typical day (circle the appropriate option):

	Yes	No
Breakfast:	1	0
Morning snack:	1	0
Lunch:	1	0
Afternoon snack/coffee:	1	0
Dinner:	1	0
Snack:	1	0
Late-evening snack/supper:	1	0
Late-night snack:	1	0

27. Where do you usually eat your hot meals on a typical weekday?

1) At home or home-prepared meals elsewhere (e.g., packed lunch)
2) Outside the home (e.g., workplace cafeteria or restaurant)
3) Both home-prepared and meals prepared outside the home

28. What kind of fat do you usually use in cooking at home?

1) We don't cook at home
2) Butter, Oivariini, or similar dairy fat product
3) Hard margarine (e.g. Becel, Flora)
4) Liquid vegetable fat product (e.g. Keiju, Flora liquid, Flora Culinesse)
5) Hard margarine used for baking (e.g. Milda, Sunnuntai)
6) Cooking oil
7) Fat is not usually used in cooking

29. What type of salad dressing do you usually choose?

1) I do not use salad dressings
2) Oil-based salad dressing or oil
3) Sour cream-based dressing
4) Mayonnaise-based dressing
5) Juice
6) Fat-free dressings

30. What kind of fat do you usually use in baking?

1) We don't bake at home
2) Butter, Oivariini, or similar
3) Hard margarine
4) Liquid vegetable fat product (bottle margarine)
5) Hard margarine used for baking (e.g. Milda, Sunnuntai)
6) Cooking oil

31. How often do you eat foods fried in fat?

1) Daily
2) 4-6 times a week
3) 1-3 times a week
4) Less than once a week

32. How often do you eat breakfast within an hour of waking up?

1) Daily
2) 5-6 times a week
3) 3-4 times a week
4) 1-2 times a week
5) Less often or never

How often do you usually eat the following foods? Please circle the most appropriate option for frequency of use. If your typical portion size is smaller than the average portion size given on the form, you may consider that by estimating your frequency slightly lower than the normal. Conversely, if your portion is larger than the stated portion size, adjust your frequency estimation to be slightly higher.

IF YOU DO NOT USE THE PRODUCT, PLEASE CIRCLE "NEVER" OR "RARELY" IN THE FORM.

DAIRY PRODUCTS

Average consumption in the last 12 months (circle one option)

Note: Coffee cream will be asked later.	Never, rarely	1-3 times/ month	Once/ week	2-4 times/ week	5-6 times/ week	Once/ day	2-3 times /day	4-5 times /day	≥ 6 times /day
**33. Whole milk** (1 glass, 1.7 dL)	0	1	2	3	4	5	6	7	8
**34. Reduced fat milk, 1.5% fat** (1 glass)	0	1	2	3	4	5	6	7	8
**35. Reduced fat milk, 1% fat** (1 glass)	0	1	2	3	4	5	6	7	8
**36. Skimmed milk** (1 glass)	0	1	2	3	4	5	6	7	8
**37. Organic skimmed milk** (1 glass)	0	1	2	3	4	5	6	7	8
**38. Organic light** milk (1 glass)	0	1	2	3	4	5	6	7	8
**39. Soy, oat, or rice milk** (1 dL)	0	1	2	3	4	5	6	7	8
**40. Buttermilk, skimmed** (1 glass, 1.7 dL)	0	1	2	3	4	5	6	7	8
**41. Organic buttermilk** (1 glass)	0	1	2	3	4	5	6	7	8
**42. Other buttermilk** (1 glass)	0	1	2	3	4	5	6	7	8
**43. Curd or yogurt, fat-free** (1 container, 2 dL)	0	1	2	3	4	5	6	7	8
**44. Curd or yogurt, other** (1 container, 2 dL)	0	1	2	3	4	5	6	7	8
**45. High-fat cheese, fat >26%, e.g. Turunmaa, Emmental** (2 slices = 20 g)	0	1	2	3	4	5	6	7	8
**46. Medium-fat cheese, fat 18-26%, e.g. Edam** (2 slices = 20 g)	0	1	2	3	4	5	6	7	8
**47. Low-fat cheese, fat 10-17%, e.g. Oltermanni 17%** (20 g)	0	1	2	3	4	5	6	7	8
**48. Cheese, fat less than 10%, e.g. Polar 5%** (20 g)	0	1	2	3	4	5	6	7	8
49. **Quark** (1 dL)	0	1	2	3	4	5	6	7	8
50. **Cottage cheese** (1 dL)	0	1	2	3	4	5	6	7	8
**51. Cream for cooking** (1 dL)	0	1	2	3	4	5	6	7	8
**52. Plant-based cream alternative for cooking** (1 dL)	0	1	2	3	4	5	6	7	8

GRAIN PRODUCTS

Average consumption in the last 12 months (circle one option)

	Never, rarely	1-3 times/ month	Once/ week	2-4 times/ week	5-6 times/ week	Once/ day	2-3 times /day	4-5 times /day	≥ 6 times /day
**53. Rye bread** (1 slice, 30 g)	0	1	2	3	4	5	6	7	8
**54. Mixed grain** bread (1 slice, 30 g)	0	1	2	3	4	5	6	7	8
**55. White bread, baguette, or other fully white bread** (1 slice or piece, 30 g)	0	1	2	3	4	5	6	7	8
**56. Crispbread, rye crisp** (1 slice)	0	1	2	3	4	5	6	7	8
**57. Porridge or gruel** (2 dL)	0	1	2	3	4	5	6	7	8
**58. Breakfast cereal** (2 dL)	0	1	2	3	4	5	6	7	8
**59. Muesli** (1 dL)	0	1	2	3	4	5	6	7	8
**60. Bun or bun-based pastry, e.g., toast or blueberry pie** (1 piece)	0	1	2	3	4	5	6	7	8
**61. Danish pastry or donut** (1 piece)	0	1	2	3	4	5	6	7	8
**62. Cake, Swiss roll, or pastry** (1 serving or 1 piece)	0	1	2	3	4	5	6	7	8
**63. Savory cracker, e.g., Cream Cracker** (2 pieces)	0	1	2	3	4	5	6	7	8
**64. Sweet cookie or biscuit** (1 piece)	0	1	2	3	4	5	6	7	8
**65. Karelian pie** (1 piece, 65 g)	0	1	2	3	4	5	6	7	8
**66. Savory pastry or pie** (1 piece, 65 g)	0	1	2	3	4	5	6	7	8

IF YOU DO NOT USE THE PRODUCT, PLEASE CIRCLE "NEVER" OR "RARELY" IN THE FORM.

SPREADABLE FATS

Average consumption in the last 12 months (circle one option)

	Never, rarely	1-3 times/ month	Once/ week	2-4 times/ week	5-6 times/ week	Once/ day	2-3 times /day	4-5 times /day	≥ 6 times /day
**67. Butter, Oivariini, or other butter-oil blend** (5 g)	0	1	2	3	4	5	6	7	8
**68. Margarine 60-70%** (5 g)	0	1	2	3	4	5	6	7	8
**69. Margarine 20-40%** (5 g)	0	1	2	3	4	5	6	7	8
**70. Benecol or Becel Pro.activ spread** (5 g)	0	1	2	3	4	5	6	7	8
**71. Processed or cream cheese, fat ≥ 20%** (e.g., Olympia, Koskenlaskija, 5 g)	0	1	2	3	4	5	6	7	8
**72. Processed or cream cheese, fat less than 20%** (e.g., Polar spreadable or Pirkka, 5 g)	0	1	2	3	4	5	6	7	8

MEAT, CHICKEN, EGGS, TOFU Average consumption in the last 12 months

	Never, rarely	1-3 times/ month	Once/ week	2-4 times/ week	5-6 times/ week	Once/ day	2-3 times /day	4-5 times /day	≥ 6 times /day
**73. Cured sausage, salami** (2 slices)	0	1	2	3	4	5	6	7	8
**74. Cold cut sausage** (2 slices)	0	1	2	3	4	5	6	7	8
**75. Liver sausage** (1 cm slice)	0	1	2	3	4	5	6	7	8
**76. Whole meat cold cut, e.g., salted meat or cooked ham** (2 slices)	0	1	2	3	4	5	6	7	8
**77. Ground meat dish, e.g., meatballs or meat sauce** (1 serving)	0	1	2	3	4	5	6	7	8
**78. Whole meat dish, e.g., steak, roast, or meat sauce** (1 serving)	0	1	2	3	4	5	6	7	8
**79. Poultry dish** (1 serving)	0	1	2	3	4	5	6	7	8
**80. Sausage dish, such as Frankfurter or baked sausage** (1 serving)	0	1	2	3	4	5	6	7	8
**81. Soup, e.g., meat or pea soup** (3 dl)	0	1	2	3	4	5	6	7	8
**82. Liver dish** (1 serving)	0	1	2	3	4	5	6	7	8
**83. Blood dish** (1 serving)	0	1	2	3	4	5	6	7	8
**84. Bacon** (5 slices, approx. 100 g)	0	1	2	3	4	5	6	7	8
**85. Pizza** (300 g serving)	0	1	2	3	4	5	6	7	8
**86. Hamburger** (regular size, approx. 150 g)	0	1	2	3	4	5	6	7	8
**87. Egg** (1 piece)	0	1	2	3	4	5	6	7	8
**88. Tofu** (100 g)	0	1	2	3	4	5	6	7	8

FISH Average consumption in the last 12 months (circle one option)

	Never, rarely	1-3 times/ month	Once/ week	2-4 times/ week	5-6 times/ week	Once/ day	2-3 times /day	4-5 times /day	≥ 6 times /day
**89. Fish dish made from the following fish: salmon, rainbow trout, trout, Arctic char, sardine, or mackerel, baked, fried, etc.** (1 serving or 1 can, approx. 150 g)	0	1	2	3	4	5	6	7	8
**90. Fish dish made from the following fish: vendace, bream, whitefish, roach, or Baltic herring, baked, fried, etc.** (1 serving or 1 can, approx. 150 g)	0	1	2	3	4	5	6	7	8
**91. Fish dish made from the following fish: saithe, perch, pikeperch, burbot, or pike, baked, fried, etc.** (1 serving or 1 can, approx. 150 g)	0	1	2	3	4	5	6	7	8
**92. Fish soup with salmon or rainbow trout** (1 serving = 3 dL)	0	1	2	3	4	5	6	7	8
**93. Fish soup with other types of fish** (1 serving = 3 dL)	0	1	2	3	4	5	6	7	8
**94. Sardine or mackerel in a can** (1 can = approx. 100 g)	0	1	2	3	4	5	6	7	8
**95. Tuna in oil** (½ can = 70 g)	0	1	2	3	4	5	6	7	8
**96. Tuna in water** (½ can = 70 g)	0	1	2	3	4	5	6	7	8
**97. Herring** (2 slices)	0	1	2	3	4	5	6	7	8
**98. Other seasoned or salted fish, such as gravlax or anchovies** (2 slices)	0	1	2	3	4	5	6	7	8
**99. Fish pie (kalakukko)** (200 g)	0	1	2	3	4	5	6	7	8
**100. Shrimp, mussels, shellfish** (1 dL)	0	1	2	3	4	5	6	7	8

IF YOU DO NOT USE THE PRODUCT, PLEASE CIRCLE "NEVER" OR "RARELY" IN THE FORM.

VEGETABLES

Average consumption in the last 12 months (circle one option)

	Never, rarely	1-3 times/ month	Once/ week	2-4 times/ week	5-6 times/ week	Once/ day	2-3 times /day	4-5 times /day	≥ 6 times /day
**101. Fresh salads** (1.5 dL)	0	1	2	3	4	5	6	7	8
**102. Grated vegetables** (1.5 dL)	0	1	2	3	4	5	6	7	8
**103. Mayonnaise-based salad, such as potato or beetroot salad** (1 dL)	0	1	2	3	4	5	6	7	8
**104. Raw root vegetables** (1 carrot or equivalent)	0	1	2	3	4	5	6	7	8
**105. Tomato** (1 piece)	0	1	2	3	4	5	6	7	8
**106. Cucumber** (2 cm piece)	0	1	2	3	4	5	6	7	8
**107. Cauliflower, fresh** (1 dL)	0	1	2	3	4	5	6	7	8
**108. Bell pepper** (2 slices)	0	1	2	3	4	5	6	7	8
**109. Beetroot, cooked** (1 dL)	0	1	2	3	4	5	6	7	8
COOKED VEGETABLES **110. Mixed vegetables, soup vegetables, wok vegetables** (1 dL)	0	1	2	3	4	5	6	7	8
**111. Peas and beans** (1 dL)	0	1	2	3	4	5	6	7	8
**112. Cauliflower** (1 dL)	0	1	2	3	4	5	6	7	8
**113. Asparagus or Brussels sprouts** (1 dL)	0	1	2	3	4	5	6	7	8
**114. Carrot** (1 dL)	0	1	2	3	4	5	6	7	8
**115. Cabbage casserole, soup, or rolls** (1 serving)	0	1	2	3	4	5	6	7	8
**116. Other vegetable dishes, such as pureed soup** (1 serving)	0	1	2	3	4	5	6	7	8
**117. Mushrooms** (1 dL)	0	1	2	3	4	5	6	7	8

POTATO, RICE, PASTA

	Never, rarely	1-3 times/ month	Once/ week	2-4 times/ week	5-6 times/ week	Once/ day	2-3 times /day	4-5 times /day	≥ 6 times /day
**118. Boiled or baked potato or mashed potatoes** (2 pieces or 2 dL)	0	1	2	3	4	5	6	7	8
**119. Fried or French fries** (2 dL)	0	1	2	3	4	5	6	7	8
**120. Potato-based casseroles or gratins** (1 serving)	0	1	2	3	4	5	6	7	8
**121. Pasta-based casseroles or lasagna** (1 serving)	0	1	2	3	4	5	6	7	8
**122. Boiled rice or risotto, rice noodles** (1 serving)	0	1	2	3	4	5	6	7	8
**123. Spaghetti or other pasta products such as wheat noodles** (1 serving)	0	1	2	3	4	5	6	7	8

FRUITS AND BERRIES

Average consumption in the last 12 months

	Never, rarely	1-3 times/ month	Once/ week	2-4 times/ week	5-6 times/ week	Once/ day	2-3 times /day	4-5 times /day	≥ 6 time s/da y
**124. Apple** (1 piece)	0	1	2	3	4	5	6	7	8
**125. Orange, grapefruit** (1 piece)	0	1	2	3	4	5	6	7	8
**126. Mandarin, satsuma, etc.** (2 pieces)	0	1	2	3	4	5	6	7	8
**127. Banana** (1 piece)	0	1	2	3	4	5	6	7	8
**128. Grapes** (10 pieces)	0	1	2	3	4	5	6	7	8
**129. Avocado** (1 piece)	0	1	2	3	4	5	6	7	8
**130. Other fruits like pear, kiwi, or melon** (1 piece or 100 g)	0	1	2	3	4	5	6	7	8
**131. Fresh or frozen berries** (1 dL)	0	1	2	3	4	5	6	7	8
**132. Berry kissel (kiisseli, berry soup)** (1 dL)	0	1	2	3	4	5	6	7	8
**133. Berry or fruit quark** (1 dL)	0	1	2	3	4	5	6	7	8
**134. Diluted berry juice** (1 glass, 1.7 dL)	0	1	2	3	4	5	6	7	8
**135. Orange juice** (1 glass)	0	1	2	3	4	5	6	7	8
**136. Apple juice** (1 glass)	0	1	2	3	4	5	6	7	8
**137. Other fruit juice** (1 glass)	0	1	2	3	4	5	6	7	8
**138. Jam or marmalade** (1 tablespoon)	0	1	2	3	4	5	6	7	8

IF YOU DO NOT USE THE PRODUCT, PLEASE CIRCLE "NEVER" OR "RARELY" IN THE FORM.

DESSERTS, SWEETS, ETC.

Average consumption in the last 12 months

	Never, rarely	1-3 times/ month	Once/ week	2-4 times/ week	5-6 times/ week	Once/ day	2-3 times /day	4-5 times /day	≥ 6 times /day
**139. Salmiakki (salty liquorice)** (1 pack or 40 g)	0	1	2	3	4	5	6	7	8
**140. Liquorice** (6 pieces or 1 piece of a 20 g bar)	0	1	2	3	4	5	6	7	8
**141. Candy** (e.g., loose sweets, 100 g)	0	1	2	3	4	5	6	7	8
**142. Ice cream or pudding** (1.5 dL)	0	1	2	3	4	5	6	7	8
**143. Chocolate** (1 large bar or 45 g)	0	1	2	3	4	5	6	7	8

DRINKS, SWEETENERS, ETC.

Average consumption in the last 12 months

	Never, rarely	1-3 times/ month	Once/ week	2-4 times/ week	5-6 times/ week	Once/ day	2-3 times /day	4-5 times /day	≥ 6 times /day
**144. Filter coffee** (1 cup, 1.5 dL)	0	1	2	3	4	5	6	7	8
**145. Boiled coffee or other coffee made without filter paper** (1 cup)	0	1	2	3	4	5	6	7	8
**146. Instant coffee** (1 cup)	0	1	2	3	4	5	6	7	8
**147. Tea** (1 cup, 1.5 dL)	0	1	2	3	4	5	6	7	8
**148. Tea-like drink, such as chamomile tea** (1 cup)	0	1	2	3	4	5	6	7	8
**149. Hot chocolate** (1 cup)	0	1	2	3	4	5	6	7	8
**150. Milk in coffee or tea** (1 tablespoon)	0	1	2	3	4	5	6	7	8
**151. Sugar in coffee or tea** (2 cubes)	0	1	2	3	4	5	6	7	8
**152. Cream in coffee or tea** (1 tablespoon)	0	1	2	3	4	5	6	7	8
**153. Sweetener in drinks or otherwise, such as saccharin**	0	1	2	3	4	5	6	7	8
**154. Honey** (1 tablespoon, 20 g)	0	1	2	3	4	5	6	7	8
**155. Sugar-sweetened soft drinks or energy drinks** (0.5 L)	0	1	2	3	4	5	6	7	8
**156. Artificially sweetened soft drinks** (0.5 L)	0	1	2	3	4	5	6	7	8
**157. Non-alcoholic beer or pilsner** (0.33 L)	0	1	2	3	4	5	6	7	8

ALCOHOL

Average consumption in the last 12 months (circle one option)

	Never, rarely	1-3 times/ month	Once/ week	2-4 times/ week	5-6 times/ week	Once/ day	2-3 times /day	4-5 times /day	≥ 6 times /day
**158. Beer, 3.5% alc** (0.5 L)	0	1	2	3	4	5	6	7	8
**159. Beer, 4.5% alc** (0.5 L)	0	1	2	3	4	5	6	7	8
**160. Beer, 5-5.5% alc** (0.5 L)	0	1	2	3	4	5	6	7	8
**161. Long drink** (0.5 L)	0	1	2	3	4	5	6	7	8
**162. Long drink, artificially sweetened, light** (0.5 L)	0	1	2	3	4	5	6	7	8
**163. Cider** (0.5 L)	0	1	2	3	4	5	6	7	8
**164. Cider, artificially sweetened, light** (0.5 L)	0	1	2	3	4	5	6	7	8
**165. Red wine** (restaurant serving, 12 cL = 1.2 dL)	0	1	2	3	4	5	6	7	8
**166. White wine** (restaurant serving, 12 cL = 1.2 dL)	0	1	2	3	4	5	6	7	8
**167. Other wine** (sparkling wine, rosé, restaurant serving, 1.2 dL)	0	1	2	3	4	5	6	7	8
**168. Strong wine** (liqueur, sherry, port wine, etc., restaurant serving 8 cL)	0	1	2	3	4	5	6	7	8
**169. Spirits or other strong alcoholic beverages** (restaurant serving 4 cL)	0	1	2	3	4	5	6	7	8

OTHER FOOD ITEMS

Average consumption in the last 12 months

	Never, rarely	1-3 times/ month	Once/ week	2-4 times/ week	5-6 times/ week	Once/ day	2-3 times /day	4-5 times/d ay	≥ 6 times /day
**170. Pickled cucumber** (5 slices)	0	1	2	3	4	5	6	7	8
**171. Salted nuts or seeds** (50 g)	0	1	2	3	4	5	6	7	8
**172. Unsalted nuts or seeds** (50 g)	0	1	2	3	4	5	6	7	8
**173. Potato chips, popcorn, or similar** (2 dL, about 20 g)	0	1	2	3	4	5	6	7	8
**174. Olives** (5 pieces)	0	1	2	3	4	5	6	7	8
**175. Fermented dairy products containing probiotics, such as Gefilus, Rela, or Actimel yogurts or drinks***	0	1	2	3	4	5	6	7	8

*** Their use should also be marked under yogurt in sections 43 - 44 and juices in sections 134 - 137 of the form.**

USE OF SALT

176. When eating outside your home, does the food generally taste

1. Less salty than your home-cooked food
2. As salty as your home-cooked food
3. Saltier than your home-cooked food

177. How do ready-made meals (store-bought prepared foods) compare to home-cooked meals in terms of salt content?

1. Less salty than your home-cooked food
2. As salty as your home-cooked food
3. Saltier than your home-cooked food
4. I do not use ready-made meals

178. Do you add salt to the food already on your plate during meals?

1. Never or very rarely
2. Sometimes, after tasting it first
3. Often, after tasting it first
4. Often, without tasting the flavor

179. The type of salt you use at home: Do you typically use

1. Regular iodized table salt (sodium chloride), e.g., iodized Jozo®
2. Non-iodized cooking salt (e.g., rock salt, pink salt)
3. Sea salt
4. Mineral salt, where part of the sodium is replaced with potassium and magnesium, such as PANSUOLA® or Seltin®

F. VITAMINS, NATURAL PRODUCTS, AND MEDICATIONS

180. Do you use any of the following products (circle the most appropriate option)?

	I do not use	Occasionally or seasonally	Almost daily
Multivitamin supplement (e.g., Multitabs)	0	1	2
Vitamin effervescent tablet (e.g., Berocca)	0	1	2
Vitamin D supplement, 5 – 10 µg	0	1	2
Vitamin D supplement, 11 – 19 µg	0	1	2
Vitamin D supplement, 20 µg	0	1	2
Vitamin D supplement, over 20 µg	0	1	2
Vitamin C supplement	0	1	2
Vitamin E supplement	0	1	2
Calcium supplement	0	1	2
Iron supplement	0	1	2
Magnesium supplement	0	1	2
Fatty acid supplement (e.g., fish oil capsules)	0	1	2

181. Do you use any natural products?

0 Never
1 Rarely (less than once a week)
2 Weekly (once or a few times a week)
3 Daily
What product(s)? ____

182. Have you ever used medication for the treatment of an illness?

0 Never
1 Yes, occasionally, last time _____ months ago in the year |___|___|___|___|
2 Yes, I use it regularly

183. What prescribed medications have you used regularly (for at least one month) in the past year?

Medication	Purpose of Use	Dosage per Day	Strength (e.g., mg)	Year Started (approx.)
				|___|___|___|___|
				|___|___|___|___|
				|___|___|___|___|
				|___|___|___|___|
				|___|___|___|___|
				|___|___|___|___|
				|___|___|___|___|

G. HEALTH STATUS

184. Have you ever had any illnesses that required hospitalization?

0 No (skip to question 185)
1 Yes, what illnesses and when (year)?

Illnesses	Year started
	|___|___|___|___|
	|___|___|___|___|
	|___|___|___|___|
	|___|___|___|___|
	|___|___|___|___|

185. Have you had seasonal influenza (a flu more severe than a common cold)?

0 No
1 Yes |___|___| times, the last time in |___|___|___|___|

186. How many colds or flu-like illnesses have you had in the last 12 months? |___|___| colds/flu

187. How many infections requiring antibiotic treatment have you had in the last 12 months? |___|___| times. Reason(s) for the antibiotic treatment: ________________________

____

188. Have you ever had a bone fracture? If yes, estimate how many.

0 No
1 Yes, |___|___| bone fractures

189. Have you been diagnosed with osteoporosis by a doctor?

0 No
1 Yes, diagnosed in |___|___|___|___|

190. Have you ever donated blood?

0 No (skip to question 192)
1 Yes, |___|___|___| times

191. Have you taken iron supplements to replace the iron lost during blood donation?

0 No
1 Yes, occasionally
2 Yes, always or almost always

192. How do you currently rate your health compared to others of your age?

0 Very poor
1 Poor
2 Average
3 Good
4 Excellent

193. How do you think your health was a year ago compared to others of your age?

0 Very poor
1 Poor
2 Average
3 Good
4 Excellent

194. How do you currently rate your functional ability compared to others of your age?

0 Very poor
1 Poor
2 Average
3 Good
4 Excellent

195. How do you think your functional ability was a year ago compared to others of your age?

0 Very poor
1 Poor
2 Average
3 Good
4 Excellent

H. PAIN

196. How often have you experienced pain in <u>the past month</u>?

1 I have not had any pain (move to question 213)
2 I have had pain less than once a week
3 I have had pain on average once a week
4 I have had pain several times a week
5 I have had pain daily or constantly

197. How long has your pain lasted?

1 Less than 3 months
2 3-6 months
3 More than 6 months

198. Rate your pain by circling the number that best describes your pain on average <u>during the past week</u> (1 = no pain; 10 = worst possible pain).

1 2 3 4 5 6 7 8 9 10
No pain **Worst possible pain**

199. Circle the number that best describes how much pain has interfered with your normal activities or work at home or outside the home <u>during the past week</u> (1 = not at all; 10 = completely).

1 2 3 4 5 6 7 8 9 10
Has not interfered **Has interfered completely**

Have you had any of the following pains during the past month?

	No	Yes, but less than daily	Yes, daily or constantly
**200. Pain on the right side of the body**	0	1	2
**201. Pain on the left side of the body**	0	1	2
**202. Pain above the waist**	0	1	2
**203. Pain below the waist**	0	1	2
**204. Neck pain**	0	1	2
**205. Pain in the chest area (front or back)**	0	1	2
**206. Shoulder pain**	0	1	2
**207. Lower back pain**	0	1	2
**208. Hip pain**	0	1	2
**209. Knee pain**	0	1	2
**210. Any other joint pain, what?** ____________________________	0	1	2
**211. Headache**	0	1	2
**212. Abdominal pain**	0	1	2

I. FUNCTIONAL ABILITY

Are you able to perform the following tasks?

	I can do it without difficulty	I can do it, but it is difficult	I cannot do it
**213. Getting dressed without help**	0	1	2
**214. Cutting your toenails**	0	1	2
**215. Climbing stairs without help (about one floor level without resting)**	0	1	2
**216. Walking about half a kilometer without resting**	0	1	2
**217. Carrying a load of about 5 kg for at least 100 meters**	0	1	2
**218. Running about 100 meters**	0	1	2
**219. Running more than half a kilometer**	0	1	2
**220. Riding a bicycle**	0	1	2
**221. Traveling by train or bus**	0	1	2
**222. Reading ordinary newspaper text (with or without glasses)**	0	1	2
**223. Hearing what is being said in a conversation between several people (with or without a hearing aid)**	0	1	2

J. FRACTURES

224. Have you had any doctor-diagnosed bone fractures during the previous 12 months?

0 **No** (skip to question 226)
1 **Yes, in which bones and when?** Please record the two most recent fractures in the spaces provided below.

***First* fracture during the previous 12 months**

Which bone was fractured?_________________

When did the fracture occur? |__|__|. |__|__| 201_

How did the fracture occur?

1 I slipped and fell
2 I tripped and fell
3 I fell down stairs
4 I fell from |___ |___ | meters
5 I fainted
6 I was in a bicycle accident
7 I was in a car accident
8 In another way, please specify: _____________

____

Where was the fracture treated?
1. Hospital, which one? ________________________
2. Health center, which one? ____________________
3. Other, where? ______________________________

Where was the fracture diagnosed?
1 In an X-ray
2 Diagnosed by a doctor in another way

***Second* fracture during the previous 12 months**

Which bone was fractured?________________

When did the fracture occur?|__|__|.|__|__|201_

How did the fracture occur?

1 I slipped and fell
2 I tripped and fell
3 I fell down stairs
4 I fell from |___ |___ | meters
5 I fainted
6 I was in a bicycle accident
7 I was in a car accident
8 In another way, please specify: _____________

____

Where was the fracture treated?
1. Hospital, which one? ______________________
2. Health center, which one? _________________
3. Other, where? ____________________________

Where was the fracture diagnosed?
1 In an X-ray
2 Diagnosed by a doctor in another way

225. Have you had more than two fractures during the previous 12 months?

0 **No**
1 **Yes**, |___|___| fractures

K. FALLS

226. How many times have you fallen in the past year (12 months), including falls that resulted in no injury?

0 I have not fallen at all (proceed to question 232)

1 I have fallen |____|____|____| times

227. When did you fall for the last time?

_____________ (month) |___|___|___|___| (year)

228. Where did you fall for the last time?

1 Inside at home
2 In the yard at home
3 On the street or road
4 Somewhere indoors
5 Somewhere outdoors

229. How did you fall for the last time?

1 I fell going upstairs
2 I fell going downstairs
3 I slipped
4 I tripped
5 I fell in another way, how? ____________________________________

230. What body part was impacted when you fell last time?

1 Head
2 Hand, upper limb
3 Leg, lower limb
4 Hip, buttock
5 Other part of the body

231. Did you visit a doctor due to your most recent fall?

0 No
1 Yes, for what injury? ____

232. Do you use fall protection equipment? (Circle the correct option)

Assistive Device	I do not use	I use
**Hip protector**	0	1
**Slip-resistant shoes or shoe brakes**	0	1
**Walking stick or walking poles**	0	1
**Walker or scooter**	0	1

Since vitamin D may have a positive effect on mental well-being, we ask you to also respond to the following questions regarding your mental health and mood.

L. WELL-BEING

How do you feel? Circle the option from 1 to 7 that best reflects your perception.

233. Up to this point, has your life been

completely without a clear purpose and goal 1 2 3 4 5 6 7 its purpose and goals have been completely clear

234. How often do you feel that you don't really care what happens around you?

very rarely or never 1 2 3 4 5 6 7 very often

235. How often are you surprised by the behavior of people you thought you knew well?

never 1 2 3 4 5 6 7 constantly

236. Have you ever felt disappointed by people you trusted?

never 1 2 3 4 5 6 7 constantly

237. How often do you feel that you are treated unfairly?

very often 1 2 3 4 5 6 7 very rarely or never

238. How often do you feel that you are in a strange situation and don't know what to do?

very often 1 2 3 4 5 6 7 very rarely or never

239. Do you feel that carrying out your daily tasks is

a source of great pleasure and satisfaction 1 2 3 4 5 6 7 painful and unpleasant

240. How often do you feel that your feelings and thoughts are all mixed up?

very often 1 2 3 4 5 6 7 very rarely or never

241. How often do you experience feelings that you wouldn't allow yourself to have?

very often 1 2 3 4 5 6 7 very rarely or never

242. Many people – even strong-willed individuals – sometimes feel like they're in the way of others. How often have you felt this way?

never 1 2 3 4 5 6 7 very often

243. When something has happened, do you usually realize afterwards that you

over- or underestimated the significance of the matter 1 2 3 4 5 6 7 saw it in the right perspective

244. How often do you feel that your daily activities are somewhat meaningless?

very often 1 2 3 4 5 6 7 very rarely or never

245. How often do you feel emotions that you cannot confidently keep under control?

very often 1 2 3 4 5 6 7 very rarely or never

M. MOOD

246. How is your mood?

1 my mood is quite bright and good
2 I am not down or sad
3 I feel down and sad
4 I am constantly down, and I cannot get over it
5 I am so depressed and low that I can no longer bear it

247. How do you feel about the future?

1 I feel hopeful about the future
2 I do not feel hopeless about the future
3 the future feels somewhat depressing to me
4 I feel that I have nothing to look forward to in the future
5 the future feels hopeless to me, and I don't believe things will get better

248. How would you say your life has gone?

1 I have succeeded quite often in life
2 I do not feel like I have failed in life
3 I feel that I have failed in my attempts more often than usual
4 my life has been just a series of failures up until now
5 I feel like I have completely failed as a person

249. How satisfied or dissatisfied do you feel?

1 I am quite satisfied with my life
2 I am not particularly satisfied
3 I do not enjoy things in the same way as before
4 I feel that I hardly get any satisfaction from anything anymore
5 I am completely dissatisfied with everything

250. How do you view yourself?

1 I feel pretty good about myself
2 I do not feel bad or worthless
3 I often feel bad and worthless
4 these days, I almost always feel worthless
5 I am completely bad and worthless

251. Do you have feelings of disappointment?

1 I am satisfied with myself and my achievements
2 I am not disappointed in myself
3 I am disappointed in myself
4 I hate myself
5 I despise myself

252. Do you have thoughts related to harming yourself?

1 I have never had suicidal thoughts
2 I do not think about or want to harm myself
3 I feel that it might be better if I were dead
4 I have specific plans for suicide
5 I would commit suicide if I had the opportunity

253. How do you feel about meeting new people?

1 I enjoy meeting and talking with people
2 I have not lost interest in other people
3 I am not as interested in other people as I used to be
4 I have almost completely lost interest and feelings towards others
5 I have lost all interest in other people and do not care about them at all

254. How do you feel about making decisions?

1 it is easy for me to make different decisions
2 I can make decisions just as well as before
3 my confidence has decreased, and I try to delay making decisions
4 I have significant difficulty in making decisions
5 I am no longer able to make decisions at all

255. How do you view your appearance and physical presence?

1 I am quite satisfied with my appearance and physical presence
2 there is nothing about my appearance that bothers me
3 I am worried that I look unappealing
4 I feel that I look unattractive
5 I am convinced that I look unattractive and repulsive

256. How is your sleep?

1 I have no difficulties with sleeping
2 I sleep as well as I did before
3 I feel much more tired upon waking up than I used to
4 I am bothered by insomnia
5 I suffer from insomnia, difficulty falling asleep, or waking up too early in the night

257. How many hours do you sleep on average per night? |____|____| hours |____|____| minutes

258. And how many hours in a 24-hour period, including nighttime and naps
|____|____| hours |____|____| minutes

259. Do you feel fatigue and exhaustion?

1 Fatigue is almost completely foreign to me
2 I do not get tired more easily than usual
3 I get tired more easily than before
4 Even a small amount of work tires and exhausts me
5 I am too tired to do anything

260. How is your appetite?

1 I have no difficulties with my appetite
2 My appetite is the same as before
3 My appetite is worse than before
4 My appetite is now much worse than before
5 I no longer have any appetite

261. Are you anxious and tense?

1 I consider myself quite calm and not easily distressed
2 I do not feel anxious or nervous
3 I become anxious and tense quite easily
4 I become especially easily distressed, anxious, or tense
5 I feel constantly anxious and distressed, as if my nerves are "worn out"

Finally, we ask you to provide any measured blood and blood pressure values, if available. If you know these values (from the latest measurement), we kindly ask you to fill them in the following table:

<table>
<tr><th></th><th>Measurement result</th><th>Year</th></tr>
<tr><td>262. Blood Sugar</td><td>|___|___| . |___| mmol/L</td><td>|___|___|___|___|</td></tr>
<tr><td>263. Total Cholesterol</td><td>|___|___| . |___| mmol/L</td><td>|___|___|___|___|</td></tr>
<tr><td>264. LDL Cholesterol</td><td>|___| . |___| mmol/L</td><td>|___|___|___|___|</td></tr>
<tr><td>265. HDL Cholesterol</td><td>|___| . |___| mmol/L</td><td>|___|___|___|___|</td></tr>
<tr><td>266. Triglycerides</td><td>|___|___| . |___|___| mmol/L</td><td>|___|___|___|___|</td></tr>
<tr><td>267. Blood Pressure</td><td>|__|__|__| / |__|__|__| mmHg</td><td>|___|___|___|___|</td></tr>
</table>

Please check once again that you have answered every question.

RETURN THIS FORM IN ITS ENTIRETY IN THE FREE POSTAGE RETURN ENVELOPE INCLUDED.

Thank you for your response!

Finnish Vitamin D Trial

N.B. The following question was asked only at the end of the study (as the last question of the study questionnaire)

During the study, considering the entire five-year period, I have regularly used the study products I received according to the instructions (circle the most appropriate option):

1) 100% or almost 100% (all)
2) 100% - 90% (more than nine out of ten)
3) 90% - 80% (more than eight out of ten)
4) 80% - 50% (more than half)
5) Less than 50% (less than half)

You may also comment on your use of the study products here:
